# Thyroid Hormones Deficiency Impairs Male Germ Cell Development: A Cross Talk Between Hypothalamic-Pituitary-Thyroid, and—Gonadal Axes in Zebrafish

**DOI:** 10.3389/fcell.2022.865948

**Published:** 2022-05-12

**Authors:** Maira S. Rodrigues, Aldo Tovo-Neto, Ivana F. Rosa, Lucas B. Doretto, Hamideh P. Fallah, Hamid R. Habibi, Rafael H. Nóbrega

**Affiliations:** ^1^ Aquaculture Program (CAUNESP), São Paulo State University (UNESP), São Paulo, Brazil; ^2^ Reproductive and Molecular Biology Group, Department of Structural and Functional Biology, Institute of Biosciences, São Paulo State University (UNESP), Botucatu, Brazil; ^3^ Department of Biological Sciences, University of Calgary, Calgary, AB, Canada

**Keywords:** hypothyroidism, methimazole, thyroid hormones, spermatogenesis, zebrafish, germ cell

## Abstract

In vertebrates, thyroid hormones are critical players in controlling different physiological processes such as development, growth, metabolism among others. There is evidence in mammals that thyroid hormones are also an important component of the hormonal system that controls reproduction, although studies in fish remain poorly investigated. Here, we tested this hypothesis by investigating the effects of methimazole-induced hypothyroidism on the testicular function in adult zebrafish. Treatment of fish with methimazole, *in vivo*, significantly altered zebrafish spermatogenesis by inhibiting cell differentiation and meiosis, as well as decreasing the relative number of spermatozoa. The observed impairment of spermatogenesis by methimazole was correlated with significant changes in transcript levels for several genes implicated in the control of reproduction. Using an *in vitro* approach, we also demonstrated that in addition to affecting the components of the brain-pituitary-peripheral axis, T3 (triiodothyronine) also exerts direct action on the testis. These results reinforce the hypothesis that thyroid hormones are an essential element of multifactorial control of reproduction and testicular function in zebrafish and possibly other vertebrate species.

## 1 Introduction

The production of thyroid hormones in fish and other vertebrates is under the control of the hypothalamic-pituitary–thyroid (HPT) axis (Cooke et al., 1994; Tousson et al., 2011; Duarte-Guterman et al., 2014; Kang et al., 2020). The thyrotropin-releasing factor [thyrotropin-releasing hormone (TRH)/corticotropin-releasing hormone (CRH)] stimulates the pituitary to release the thyrotropic hormone (TSH), which in turn, promotes the synthesis and release of thyroid hormones, thyroxine (T4) and triiodothyronine (T3), by thyroid follicles (Larsen et al., 1998). Of the two, T3 is the more biologically active thyroid hormone owing to its affinity for the nuclear thyroid hormone receptor (Carr and Patiño, 2011). The HPT axis acts parallel to the hypothalamic-pituitary–gonadal (HPG) axis, which involves a large number of hormones, including the gonadotropin-releasing hormone (GnRH) that promotes the secretion of gonadotropin hormones, follicle-stimulating hormone (FSH) and luteinizing hormone (LH) (Schulz et al., 2010; Pankhurst, 2016) which are crucial for testis development and spermatogenesis in fish (Schulz et al., 2010; for a review see Xie et al., 2020). There is evidence for interaction between HPT and HPG axes in vertebrates (Teerds et al., 1998; Ariyaratne et al., 2000; Matta et al., 2002; Wagner et al., 2009; Morais et al., 2013; Kang et al., 2020), although this subject remains poorly investigated in fish, particularly, in the male reproductive system (Habibi et al., 2012; Castañeda-Cortés et al., 2014; Tovo-Neto et al., 2018; Ma et al., 2020a; Ma et al., 2020b). In mammals, it has been shown that T3 regulates the growth and maturation of testis by inhibiting immature Sertoli cell proliferation and stimulating their terminal differentiation (Cooke and Meisami, 1991; Hess et al., 1993; Cooke et al., 1994). Furthermore, in postnatal rat testis, an important action of thyroid hormones is to initiate the onset of Leydig cell differentiation and stimulation of steroidogenesis (Ariyaratne et al., 2000), in part, by stimulating the expression of steroidogenic acute regulatory protein (StAR) (Mendis-Handagama and Ariyaratne, 2005). The presence of thyroid hormone receptors in the mammalian testis, particularly in Leydig cells, suggests both direct and indirect actions of thyroid hormones on testicular function (Hernandez, 2018). The regulatory role of thyroid hormones is complex, species specific, and dependent on developmental stages. Neonatal hypothyroidism was shown to impair testicular growth and sperm production in rats (França et al., 1995), hamsters (*Mesocricetus auratus*) (Jansen et al., 2007), and juvenile teleost fish (*Oreochromis niloticus*) (Matta et al., 2002).

A number of researchers have investigated the role of thyroid hormones in fish embryogenesis, larval development, and growth (Blanton and Specker, 2007; Mukhi et al., 2007; Orozco et al., 2012). With regards to fish male reproduction, there are some evidence that thyroid hormones can affect spermatogenesis (Cyr and Eales, 1996; Wagner et al., 2009; Nelson et al., 2010; Habibi et al., 2012; Morais et al., 2013; Safian et al., 2016; Tovo-Neto et al., 2018). In adult catfish, *Clarias gariepinus*, treatment with thiourea (a thyroid disruptor) decreased 11-ketostestosterone (11-KT) and testosterone (T) production (Swapna et al., 2006), leading to male reproductive system disruption. Morais and collaborators (2013) demonstrated the influence of thyroid hormones on zebrafish spermatogenesis using an *ex vivo* approach. In the same study, the authors revealed that T3 stimulated the increase in mitotic index of type A undifferentiated spermatogonia (A_und_) and Sertoli cells through Igf3 (Insulin-like growth factor like 3), a Sertoli cell stimulatory growth factor (Morais et al., 2013). Moreover, the same authors showed that T3 potentiated FSH actions on steroid release and enhanced Fsh-stimulated *cyp17a1* (17α-hydroxylase/17, 20 lyase) and *ar* mRNA levels in adult zebrafish testis (Morais et al., 2013). There is also evidence that thyroid hormones interact with other reproductive peptides such as GnRH and gonadotropin-inhibitory hormones (GnIH) *in vivo* (Ma et al., 2020a, Ma et al., 2020b) and *in vitro* in the zebrafish testis (Rodrigues et al., 2021). Other studies have demonstrated that GnRH stimulates thyroid activity in a freshwater murrel, *Channa gachua*, and two carp species, *Catla* and *Cirrhinus mrigala* (Roy et al., 2000). GnRH injection increases plasma T4 levels in different species (Jacobs et al., 1988; Roy et al., 2000; Chiba et al., 2004), suggesting an effect of endogenous pituitary gonadotropin release due to the heterothyrotropic activities of GnRH on the thyroid (Mackenzie, 1982, Mackenzie et al.,^1987^). In general, these findings support the hypothesis that normal thyroid hormone action is critical for HPG axis function and normal gonadal function. However, significant gaps remain regarding exact physiological significance of thyroid hormones on male fish reproductive function.

The aim of this research was to further explore the influence of thyroid hormones on male zebrafish reproduction adopting *in vivo* and *ex vivo* approaches. We first evaluated the effect of hypothyroidism induced by methimazole and co-treatment with T4 on zebrafish spermatogenesis by histomorphometrical measurement and measured testicular transcript levels for genes related to reproduction, as well as 11-KT plasma levels and basal and FSH-induced 11-KT release *in vitro*. Subsequently, we investigated the hypothyroidism induced by methimazole treatment and T3 injection on zebrafish brain and pituitary by transcript measurement. Finally, we investigated long-term effects of T3 on zebrafish spermatogenesis by histomorphometrical analysis, and transcript levels of a selected genes. The results provide a framework for understanding of the influence of thyroid hormone in the control of male reproduction in adult zebrafish.

## 2 Materials and Methods

### 2.1 Animals

Sexually mature male zebrafish (outbred) (4–5 months-old) were maintained in the aquarium facility of the Department of Structural and Functional Biology, Institute of Biosciences, Botucatu, São Paulo State University (UNESP) in 6-L tanks in the recirculating system under constant temperature conditions (28°C), and proper photothermal conditions (14-h light/10-h dark). The following water parameters were monitored in all tanks every other day: pH, salinity, dissolved oxygen, and ammonia concentration. The animals were fed twice a day with commercial food (Sera Vipan Flakes^®^). Handling and experimentation were performed according to the Brazilian legislation regulated by National Council for the Control of Animal Experimental (CONCEA) and Ethical Principles in Animal Research (Protocol n. 1031-CEUA) and University of Calgary animal care committee and in agreement with the procedures of the Canadian Council of Animal Care (Protocol #AC19-0161).

### 2.2 Treatment Solutions: Methimazole-Induced Hypothyroidism and Reversal Treatment With T4

In this study, we used methimazole (1-methyl-3H-imidazole-2-thione) (CAS 60-56-0; MW, 114.17 g/mol; purity, ≥99%; Sigma-Aldrich, St. Louis, MO, United States) to chemically generate hypothyroidism in adult zebrafish males. Exposure concentration of 1 mM was prepared following the methodology described in Rodrigues et al. (2021). The working concentration of 1 mM methimazole and 100 μg/L T4 (L-Thyroxine) (CAS 51-48-9; MW, 776.87 g/mol; purity ≥98%; Sigma-Aldrich, St. Louis, MO, United States) were chosen based on previous studies (Sharma and Patiño, 2013; Sharma et al., 2016; Rodrigues et al., 2021). In this study, adult male zebrafish (*n* = 144) were divided into four replicate tanks per experimental group: control [only filtered water (*n* = 36)]; group I [filtered water with T4 (100 μg/L) (*n* = 36)]; group II [filtered water with methimazole (1 mM) (*n* = 36)]; group III [1 mM of methimazole followed by addition of T4 (100 μg/L) (*n* = 36) as reversal treatment group (methimazole + T4)]. In the T4 group, males were exposed to T4 (100 μg/L) from the second week until the end of treatment. In the methimazole group, males were exposed to 1 mM methimazole for 21 days. In addition, zebrafish males were exposed to 1 mM methimazole for 21 days, and T4 (100 μg/L) was added in the water from the second week until the end of treatment. The reversal treatment was performed to assess whether the apparent effects were due to lowering thyroid hormone levels. After euthanasia, plasma T3 levels were measured on the treatments and the heads were sampled for histology (control, methimazole and methimazole + T4), while the testes were dissected and immediately used for *in vivo* experiments (histomorphometric analysis and gene expression); androgen plasma levels and *ex vivo* organ culture experiment [short-term (18 h) incubation for androgen release by zebrafish testicular explants] were available on the treatments (control, T4, methimazole and methimazole + T4 groups). The brain and pituitary from the control and methimazole groups were sampled for gene expression.

### 2.3 Thyroid Hormone Extraction and Measurement

Blood from adult male zebrafish were collected in different conditions (control, methimazole and methimazole + T4) (*n* = 5 per condition) to confirm the hypothyroidism status. Animals were euthanized, and the caudal peduncle was cut for blood sampling using heparinized syringes and tubes. Plasma fractions were isolated after blood centrifugation at 4°C for 10 min at 800 × *g* (Eppendorf Centrifuge 5424 R) for thyroid hormone analysis. Plasma Triiodothyronine (T3) levels were measured by Enzyme-Linked Immunosorbent Assay (Competitive ELISA kit) (Invitrogen, TX061-1 EA, Carlsbad, CA, United States). This assay is designed to detect and quantify the levels of T3 in different sample types such as serum, urine, and plasma. Here, we prepare plasma sample according to the manufacturer’s procedure. Briefly, high binding 96-well strip-well plate was coated with donkey anti-sheep IgG. Plasma samples were extracted with ethyl acetate (5:1) (v/v) solvent: sample ratio. Samples were frozen and solvent solution was collected (this step was repeated for maximum extraction); and dry pooled solvent extracts down in a speedvac for 2–3 h. Then, samples were reconstituted at room temperature in the 1X Assay Buffer, and 100 μl of either standards or samples were added to the wells in duplicate. 25 μl of T3 conjugated and T3 antibody were added to each well. Plate was incubated for 2 h, shaking, at room temperature. The plate was washed with 1X Wash Buffer, followed by the addition of the detection reagent (TMB substrate solution). After, 30 min, the reaction was stopped with 1 M HCL, CAUSTIC and read at 450 nm using a microplate reader (Epoch, Agilent, Santa Clara, CA, United States). Data were evaluated as nanograms of Triiodothyronine (T3) per milliliter of plasma.

### 2.4 Thyroid Follicles Histology

Thyroid follicles from the different experimental groups were analyzed histologically. As thyroid follicles in fish appear distributed among the afferent branchial arterioles (Patinõ et a., 2003; Van der Ven et al., 2006), head was separated from the trunk and fixed in 2% glutaraldehyde and 4% paraformaldehyde in Sorensen buffer [0.1 M, pH 7.2] for at least 24 h at room temperature. The material was dehydrated, embedded in glycol methacrylate (GMA) resin (Technovit 7100 - Heraeus Kulzer, Wehrheim, Germany), and serial sections (3 μm thickness) were stained with 0.1% toluidine blue in 1% sodium borate and examined and documented using a Leica DMI6000 microscope (Leica, Heidelberg, Germany).

### 2.5 Histomorphometrical Evaluation of Zebrafish Spermatogenesis

After exposure to methimazole or methimazole + T4, zebrafish testes (*n* = 8 per treatment) were dissected and immediately fixed in 2% glutaraldehyde and 4% paraformaldehyde in Sorensen buffer [0.1 M, pH 7.2] at 4°C overnight. Subsequently, testes were dehydrated, embedded in GMA resin (Technovit 7100—Heraeus Kulzer, Wehrheim, Germany), sectioned at 3 μm thickness, and stained with 0.1% toluidine blue. The slides were evaluated, and the proportion of section surface area of spermatogenic cysts containing different germ cell types were determined: type A undifferentiated spermatogonia (A_und*_ and A_und_), type A differentiated spermatogonia (A_diff_), type B spermatogonia (SpgB), spermatocytes (Spc), and spermatids (Spt). Intersection points were counted on the histologic fields, for which five fields per slide (*n* = 8 slides per treatment) were quantified using a grid of 540 (54 × 10) intersections under 100x objective lens. The proportion of section area occupied by spermatogenic cysts containing different germ cell types were represented as fold-change of control value.

For the quantification of the relative number of spermatozoa, twenty different histological fields were captured at 100x objective lens and analyzed by IMAGEJ Software (available at http://imagej.nih.gov/ij/index.html) according to Fallah and collaborators (2019, 2020), Tovo-Neto and collaborators (2020) and Rodrigues and collaborators (2021).

### 2.6 Transcript Analysis by Quantitative Real-Time PCR (qPCR)

Total RNA from testes (control, methimazole, and methimazole + T4 groups) was extracted using TRIzol™ (Invitrogen, Carlsbad, CA, United States), according to the manufacturer’s instructions, and quantity and purity were checked with a NanoDrop™ One Spectrophotometer (Thermo Scientific, Madison, WI, United States). cDNA synthesis was performed as described previously (Nóbrega et al., 2010). qPCR reactions were conducted using 5 μL of 2x SYBR-Green Universal Master Mix, 1 μL of forward primer (9 mM), 1 μL of reverse primer (9 mM), 0.5 μL of DEPC water, and 2.5 μL of cDNA. The relative mRNA levels of *thrα* and *thrβ* (thyroid hormone receptors), *fshr* (follicle-stimulating hormone receptor), *cyp17a1* (17α-hydroxylase/17,20 lyase/17,20 desmolase), *insl3* (insulin-like peptide 3), *cx43* (testicular connexin), *igf3* (insulin-like growth factor 3), *amh* (anti-Müllerian hormone), *gsdf* (gonadal somatic cell derived factor), *nanos2* (marker of undifferentiated spermatogonia), *dazl* (deleted in azoospermia-like), *sycp3l* (synaptonemal complex protein 3) and *odf3a* (outer dense fiber protein 3) were measured in the different treatments.

Brain (*n* = 8) and pituitary (*n* = 4 pools of 4 pituitaries for each pool) were collected from control and methimazole groups. Brain of each fish was kept separate. Total RNA was extracted from the brain using TRIzol™ (Invitrogen, Carlsbad, CA, United States) method. At least four pituitary glands were pooled per group (*n* = 4 pools per treatment), and total RNA was extracted using a commercial kit (PureLink^TM^ RNA Mini Kit, Ambion, Life Technologies, Carlsbad, CA, United States). After RNA extraction, the usual downstream methods were followed according to procedures described above. The relative mRNA levels of *gnrh2* and *gnrh3* (gonadotropin-releasing hormones), *gnih* (gonadotropin-inhibitory hormone), and *crf* (corticotropin-releasing hormone) were analyzed in the brain, and the *lhb* (luteinizing hormone), *fshb* (follicle-stimulating hormone), and *tsh* (thyroid-stimulating hormone) mRNA levels were determined in the pituitary gland. mRNA levels of the targets (Cts) were normalized by the transcript levels of *β-actin* and expressed as relative values of the control group. Primers were designed according to zebrafish sequences (Supplementary Table S1).

### 2.7 11-KT Plasma Levels

Blood from adult male zebrafish in different conditions (control, T4, methimazole, and methimazole + T4 groups) were collected (*n* = 8 per condition). Fish were euthanized, and the caudal peduncle was cut for blood sampling using heparinized syringes and tubes. Subsequently, blood was centrifuged at 4°C for 10 min at 800 ×*g* (Eppendorf Centrifuge 5424 R), and 11-Ketotestosterone (11-KT) plasma levels were quantified by ELISA (582751, Cayman Chemical, Ann Arbor, MI, United States), following the manufacturer’s instructions. The results were evaluated as nanograms of 11-KT per milliliter of plasma.

### 2.8 Testis Tissue Culture

An *ex vivo* testis culture system described previously (Leal et al., 2009) was used to culture zebrafish testes. For short-term incubations (18 h for 11-KT release analysis), testes were submerged in a culture medium, whereas for long-term exposure (7 days for histomorphometrical analysis and gene expression), testes were placed on a nitrocellulose membrane on top of a cylinder of agarose (1.5% w/v, Ringer’s solution, pH 7.4) and exposed to 1 ml of medium culture in 24-well flat-bottom plates, as described by Leal and collaborators (2009).

### 2.9 Short-Term (18 h) Incubation

Zebrafish testes were collected from eight animals per condition (control, T4, methimazole, methimazole + T4) post-dissection. One testis was cultivated in the Lebovitz medium (L-15), whereas its contra-lateral one in L-15 containing recombinant zebrafish Fsh (rzfFsh; 100 ng/ml). The rzfFsh protein was obtained from ImmunoPrecise Antibodies (Europe) B.V. Science Park Utrecht, Netherlands. Following incubation, testes were individually weighed, and the medium was collected and stored at −20°C for androgen release (11-KT) assay (see Sub-Section 2.9).

### 2.10 *In Vitro* 11-KT Release by Zebrafish Testicular Explants in Short-Term Incubation

This technique was used to examine whether treatment conditions (T4, methimazole, methimazole + T4) modulated rzfFsh (100 ng/ml)-induced androgen release by zebrafish testis. The androgen (11-KT) release capacity of zebrafish testes into culture medium was measured after 18 h incubation as described previously (García-López et al., 2010). The levels of 11-KT released by zebrafish were quantified by ELISA using a commercial kit (Cayman Chemical) following manufacturer’s instructions.

### 2.11 Long-Term (7 days) Incubation

To examine the effects of 100 nM T3 (3,3’,5- Triiodo-L-Thyronine) (CAS 6893-02-3; MW, 650.97 g/mol; purity ≥95%; Sigma-Aldrich, St. Louis, MO, United States) (*n* = 8) on zebrafish spermatogenesis, long-term incubations were performed according to Leal and collaborators (2009). For that, one testis was incubated in the presence of T3 alone and its contralateral one in a basal culture medium. The proportion of section area occupied by different germ cell types were represented as fold-change of basal value. The relative number of spermatozoa per area was quantified as described above (see Sub-Section 2.4). Also, this technique was used to analyze if different concentrations of T3 and T4 (10, 100 and 1,000 nM) modulate expression of selected genes in zebrafish testis. For that, total RNA from testis explants (*n* = 8) was extracted and the relative mRNA levels of *nanos2*, *sycp3l*, *3β-HSD* (3-beta (β)-hydroxysteroid dehydrogenase), and *cyp17a1* were evaluated as described above (see Sub-Section 2.5) (Supplementary Table S1).

### 2.12 T3 Injections

In this study, we used T4 for the long-term experiments as the hormone is converted into T3 over time (see above), while to assess the rapid effects of thyroid hormones (short-term experiments), we used the active hormone (T3). Therefore, adult zebrafish were intracelomically injected with 0 and 250 ng of T3 per fish (*n* = 16). The dose was selected according to its ability to stimulate deiodinase type 3 mRNA levels as described previously (Nelson and Habibi, 2008; Nelson et al., 2010). A stock solution of T3 was dissolved in sodium hydroxide (0.02 M) and further diluted in physiologic saline. The control group was injected only with the physiologic saline solution. After 12 h, the animals were euthanized, and brain and pituitary glands were sampled. Brain of each fish (*n* = 8) was kept separate. The pituitaries were pooled (*n* = 4 pools of 4 pituitaries per condition) for RNA extraction. mRNA levels of *gnrh2* and *gnrh3*, *gnih*, and *crf* were quantified in the brain, and *lhb*, *fshb*, and *tsh* were quantified from pituitary glands (Supplementary Table S1). RNA extraction and downstream procedures were followed as described in Sub-Section 2.5 (see the Figure below).

### 2.13 Statistical Analysis

All data were subjected to normality Shapiro-Wilk test followed by the Bartlett homogeneity variance test. Significant differences between two groups were identified using unpaired or paired t-tests, while for three or more groups, one-way ANOVA followed by the Student–Newman–Keuls or Dunnett’s tests was used. Significance level (*p*) was considered ≤0.05 in both cases. Data are represented as mean ± SEM (Standard Error of Mean). All statistical analysis was performed using Graph Pad Prism software 7.04 (*Graph Pad Software*, Inc., San Diego, CA, United States, http://www.graphpad.com).

## 3 Results

### 3.1 Plasma Thyroid Hormone (T3) Levels

**FIGURE 1:**
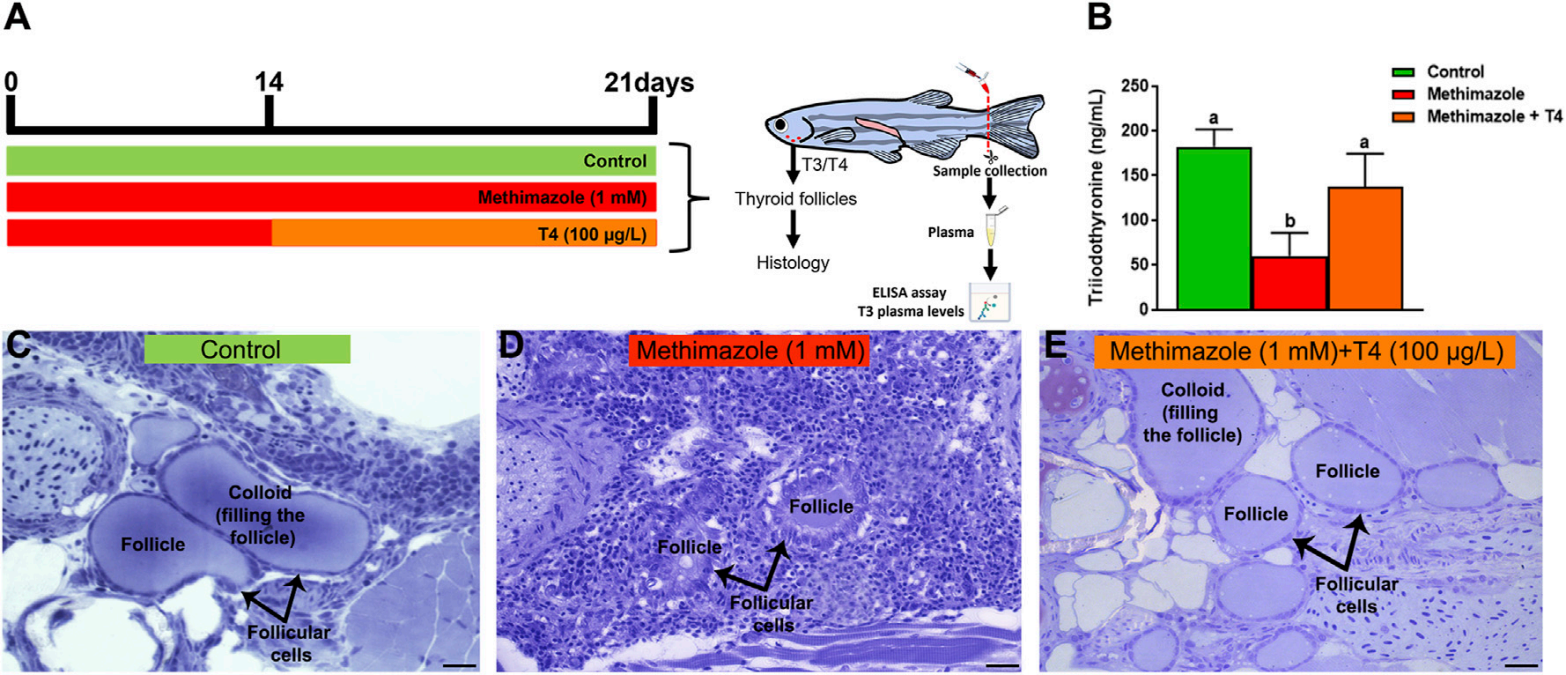
**(A)** Experimental design representation of treatments: control (non-treated fish), methimazole (1 mM) and methimazole (1 mM) co-treated with T4 (100 μg/L**)** groups. Zebrafish adult males were exposed to reconstituted water containing 1 mM methimazole (goitrogen) for 21 days or 1 mM methimazole following T4 (100 μg/L) added in the water from the second week until the end of exposure. The control group received the same volume of vehicle solution. T3 levels (ng/mL plasma) and thyroid follicles were evaluated after treatments. **(B)** Levels of Triiodothyronine (T3) in the plasma of adult male zebrafish following methimazole induced-hypothyroidism and methimazole co-treated with T4. Bars represent the mean ± SEM (*n* = 5 per condition). ANOVA followed by Dunnett’s multiple comparison tests. Distinct letters denote significant differences (*p <* 0.05) between different treatment conditions with the control group. After 3 weeks heads were dissected for histological analysis of thyroid follicles **(C–E)**. Control group **(C)** have thyroid follicles typical of euthyroid animals. These thyroid follicles display squamous or cuboidal epithelial cells and are totally filled with colloid. However, animals treated with methimazole **(D)** revealed disturbed thyroid follicles with columnar epithelium, follicle cell hypertrophy and colloid depletion, while fish co-treated with T4 **(E)** showed follicles similar to the control animals. Staining: Toluidine blue with sodium borate. Scale bar = 20 μm.

To confirm that basal thyroid hormone levels were affected after methimazole or methimazole + T4 treatment, plasma samples were collected and T3 levels were measured in the different experimental groups (Figure 1). The analysis showed that plasma T3 levels were significantly decreased following methimazole treatment (approximately 59 ng/ml) when compared to control group (approximately 182 ng/ml) (Figure 1B). In contrast, the observed effect on T3 levels after methimazole treatment was recovered by co-treatment with T4, and plasma T3 levels (approximately 137 ng/ml) were significantly similar to the levels found in control animals (Figure 1B).

### 3.2 Thyroid Follicles Analysis

Thyroid gland follicles were examined histologically in the control group and following treatments with methimazole and methimazole + T4 (Figures 1A,C,D,E). The control group thyroid follicles consisted of squamous or cuboidal epithelial cells filled with colloid (Figure 1C). The results demonstrate a significant modification in the histological condition of thyroid follicles in fish treated with methimazole. Three-week exposure to 1 mM methimazole resulted in thyroid gland follicles with colloid depletion, columnar epithelium and follicle cell hypertrophy (Figure 1D). These morphological changes were consistent with previous studies in which adult male zebrafish were exposed to methimazole (Rodrigues et al., 2021), and other goitrogens, such as perchlorate (Patiño et al., 2003) and 6-n-propyl-2-thiouracil (PTU) (Van der Ven et al., 2006). The observed effect of methimazole was reversed by co-treatment with T4, in which the thyroid follicles were found to be morphologically similar to the control group (Figure 1E). The results demonstrate that methimazole-induced hypothyroidism in zebrafish had altered thyroid function following treatment with the goitrogen. Also, the results demonstrate that co-treatment with T4 restored the zebrafish thyroid follicular structure.

### 3.3 Methimazole-Induced Hypothyroidism and Reversal Treatment With T4: Histomorphometrical Analysis of the Zebrafish Testis

**FIGURE 2:**
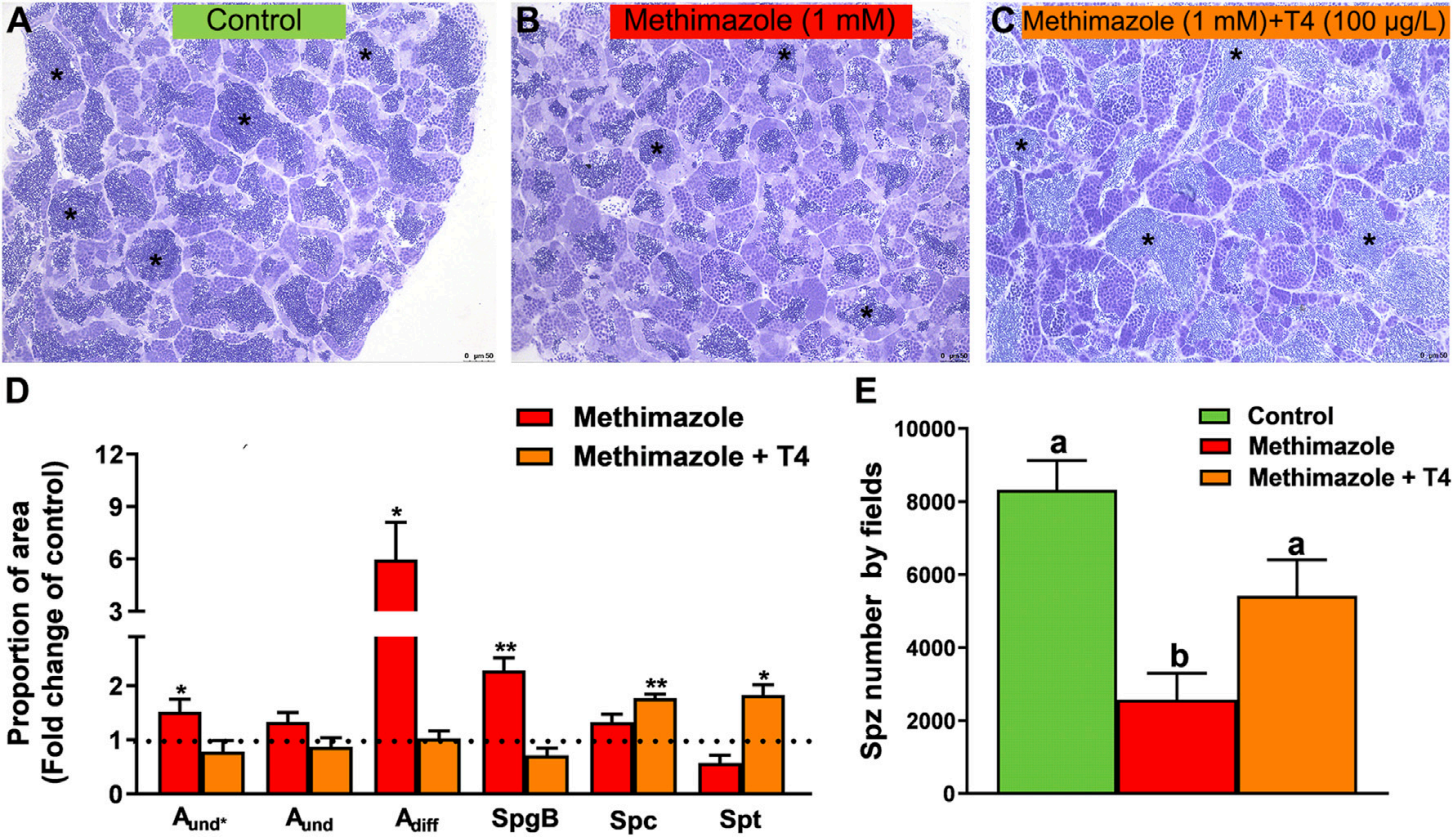
Histomorphometrical evaluation of zebrafish testes after *in vivo* exposure to methimazole and co-treatment with T4 for 3 weeks. Control group (non-treated fish) **(A)**. Methimazole-treated group **(B)**. Methimazole co-treated with T4 **(C)**. Asterisks in **(A)**, **(B)** and **(C)** indicate the testicular lumen with spermatozoa that appeared reduced in the methimazole group. **(D)** Proportion of section area occupied by different spermatogenic cysts: type A undifferentiated spermatogonia (A_und*_, A_und_), type A differentiated spermatogonia (A_diff_), type B spermatogonia (SpgB), spermatocytes (Spc), and spermatids (Spt). Bars (mean ± SEM; *n* = 8) are expressed as fold-change relative to the no-treated fish (control group) (dotted black line set at 1). **(E)** Spermatozoa number per field generated by using IMAGEJ Software from control and treatments. ANOVA followed by Dunnett’s multiple comparison tests. Distinct letters denote significant differences (*p <* 0.05) between different treatment conditions with the untreated group. Asterisks denote statistical significance differences between control, methimazole and methimazole + T4 groups; *p < 0.05; **p < 0.01 (Student unpaired *t*-test; *n* = 8). Staining: Toluidine blue. Scale bar = 50 µm.

Methimazole-induced hypothyroidism promoted histomorphometrical alterations in the proportion of germ cell cysts compared to the control (Figures 2A–D). There was a significant increase in the proportion of the area occupied by type A undifferentiated spermatogonia (A_und*_), type A differentiated (A_diff_) and spermatogenic cysts containing type B spermatogonia (SpgB) in the methimazole group as compared to control (Figure 2D). The number of meiotic cells (Spc) and post-meiotic haploid cell population (Spt) did not change between control and methimazole-induced hypothyroidism group (Figure 2D). However, the relative number of spermatozoa reduced drastically when compared to control as clearly evidenced in the photomicrographs and analysis of spermatozoa number by field (Figures 2A,B,E). Co-treatment with T4 rescued the proportion of A_und*_, A_diff_ and SpgB types returned to its basal values, while the proportion area occupied by Spc and Spt significantly increased (Figure 2D). Remarkably, the production of spermatozoa was recovered in the co-treatment with T4 (as viewed in the fields of Figures 2A,C,E).

### 3.4 Methimazole and Co-Treatment With T4: Testicular Transcript Levels

**FIGURE 3:**
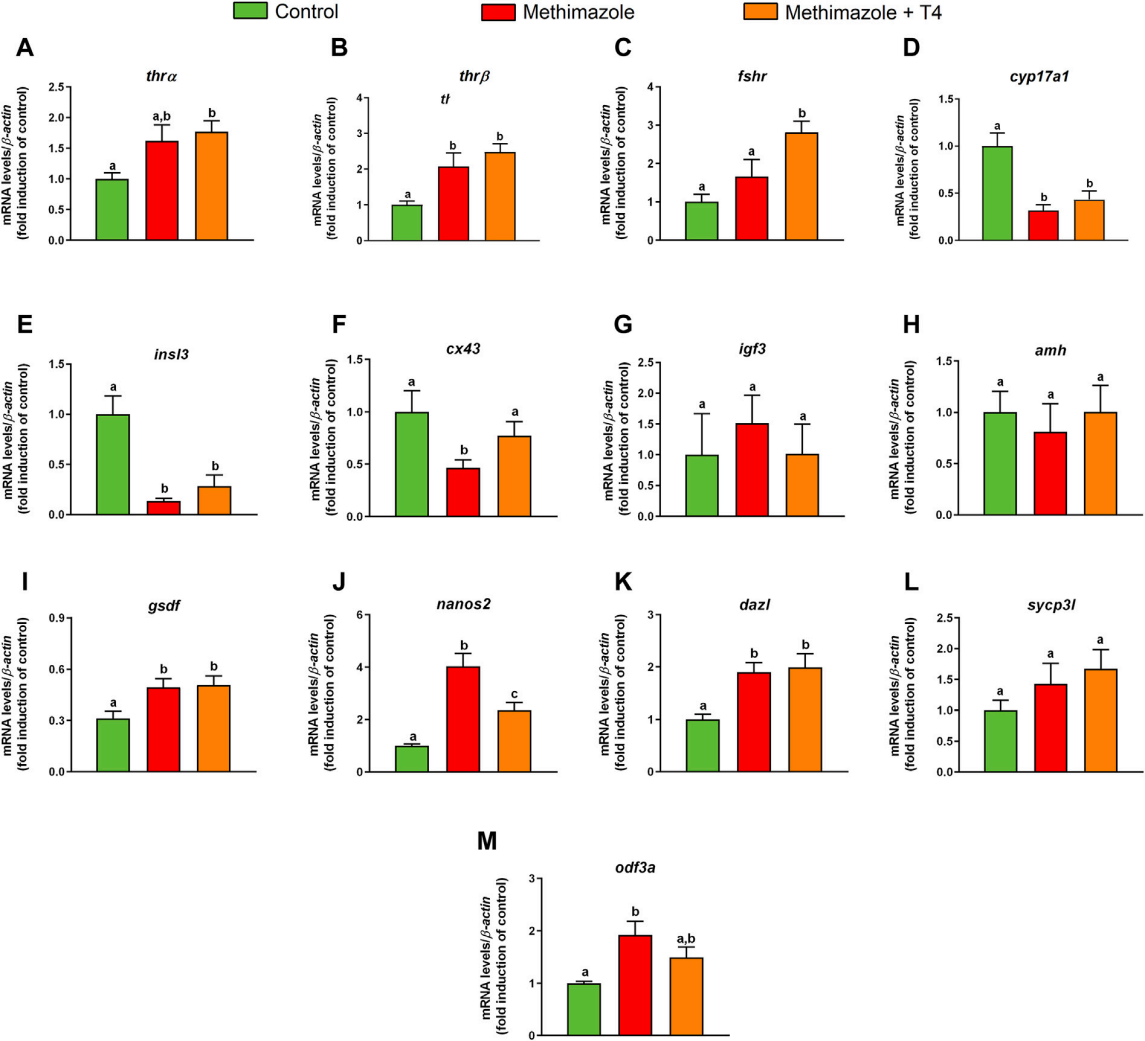
Relative mRNA levels of selected genes expressed in zebrafish testis after *in vivo* exposure to methimazole and methimazole co-treated with T4 for 3 weeks. The selected genes *thrα* and *thrβ* (thyroid hormones receptor) **(A,B)**; *fshr* (follicle-stimulating hormone receptor) **(C)**; genes expressed by somatic cells (Leydig and Sertoli cells) **(D–I)**; *cyp17a1* (17α-hydroxylase/17,20 lyase) **(D)**; *insl3* (insulin-like peptide 3) **(E)**; *cx43* (testicular connexin) **(F)**; *igf3* (insulin-like growth factor 3) **(G)**; *amh* (anti-Müllerian hormone) **(H)**; *gsdf* (gonadal somatic cell derived factor) **(I)**; and germ cell markers **(J–M)**; *nanos2* **(J)**; *dazl* (deleted-in azoospermia-like) **(K)**; *sycp3l* (synaptonemal complex protein 3) **(L)**; *odf3a* (outer dense fiber of sperm tails 3B) **(M)** were evaluated. Ct values were normalized with *β-actin* and expressed as relative values of control levels of expression. Bars represent the mean ± SEM fold change (*n* = 8) relative to the control, which is set at 1. Student unpaired *t*-test. Different letters denote significant differences (*p <* 0.05) between different treatment conditions with the control.

Transcript levels of selected genes involved in reproduction were measured by qPCR in the testis from methimazole-induced hypothyroidism and rescued groups (methimazole + T4) (Figure 3). In this study, we measured transcript levels of two thyroid hormone receptor subtypes (*thrα, thrβ*). The *thrα* transcript level was higher in the methimazole-treated group than control, but the difference was not statistically significant (Figure 3A). The *thrα* transcript level was further increased significantly in the methimazole + T4 treated group (Figure 3A). Similarly, the *thrβ* was increased in the methimazole and methimazole + T4 treated groups, compared to the control (Figure 3B).

We also measured mRNA levels of *fshr* which was not altered in the methimazole treated group, but considerably increased in the methimazole + T4 treated fish, compared to the control (Figure 3C).

In the present research, we quantified transcript levels for *cyp17a1* and insulin-like peptide 3 (*insl3*) genes. Treatment with methimazole significantly reduced the *cyp17a1* and *insl3* transcript levels compared to control (Figure 3D,E). Co-treatment with T4 did not influence the methimazole induced response on the expression of these transcripts (Figure 3D,E). We also observed significant reduction in the testicular connexin (*cx43*) mRNA levels in the methimazole treated group, compared to control (Figure 3F). Co-treatment with T4 in this case increased the *cx43* mRNA to a level not significantly different from the control (Figure 3F).

Among others, *igf3*, *amh*, and *gsdf* genes are known to be expressed in the Sertoli cells. Treatment with methimazole or methimazole + T4 were without significant effects on *igf3* and *amh* transcript levels (Figure 3G,H). The *gsdf* mRNA level, however, was higher in the methimazole and methimazole + T4 treated groups (Figure 3I).

With regard to germ cell marker genes, such as marker for type A undifferentiated spermatogonia*, nanos2* mRNA level was considerably higher in the methimazole-treated group compared to control (Figure 3J). Co-treatment with T4, significantly reduced the methimazole-induced response to a level higher than the basal control (Figure 3J). The *dazl* (deleted-in azoospermia-like) transcript level expressed by A_diff_ and SpgB, increased in the methimazole treated group compared to the control (Figure 3K). Co-treatment with T4 did not influence the methimazole-induced response (Figure 3K). In our study, synaptonemal complex protein 3 (*sycp3l*) which is a marker for spermatocytes was not significantly affected by methimazole or methimazole + T4 treatments (Figure 3L). However, the transcript level of the outer dense fiber protein 3 (*odf3a*) which is a marker for spermatids was increased following treatment with methimazole (Figure 3M). Co-treatment with T4 reduced the methimazole-induced response to a level not significantly different from the basal control (Figure 3M).

### 3.5 Measurement of 11-KT Levels

**FIGURE 4:**
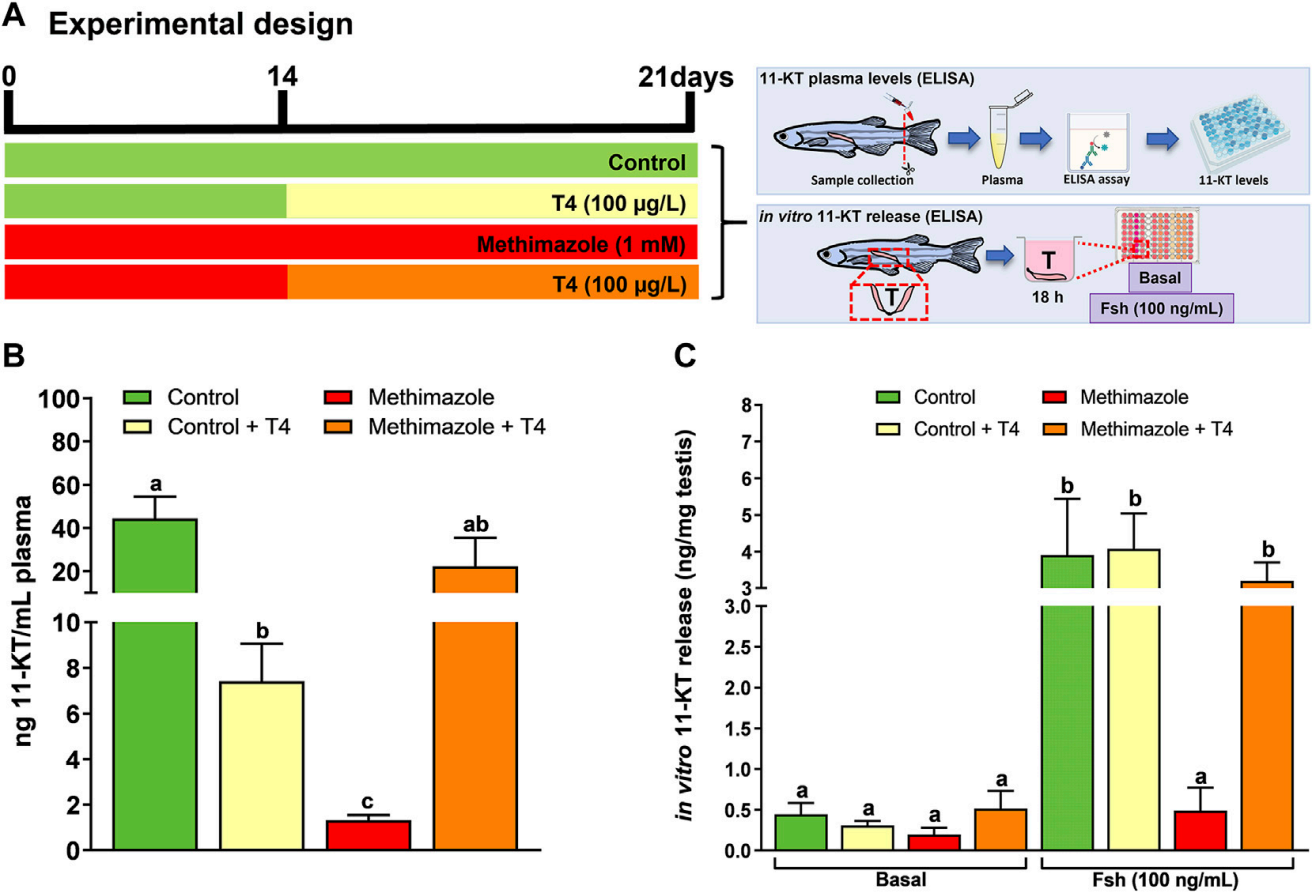
**(A)** Experimental design. 11-Ketotestosterone (11-KT) plasma levels (ng/mL plasma) was evaluated in zebrafish males exposed to different treatments: Control (non-treated fish), T4 (100 μg/L), methimazole (1 mM) and methimazole (1 mM) + T4 (100 μg/L). Amounts of 11-KT (ng/mg of testis weight) released by zebrafish testes were measured in the incubation media after 18 h (short-term exposure) in the presence or absence of Fsh (100 ng/ml) from control, T4, methimazole and methimazole + T4 groups. **(B)** Effect of T4, methimazole and combination of methimazole and T4 on 11-KT plasma levels. **(C)** Androgen (11-KT) release from zebrafish testicular explants previously treated with T4, methimazole or methimazole co-treated with T4. Bars represent the mean ± SEM (*n* = 8). ANOVA followed by Tukey’s test. Different letters denote significant difference (*p <* 0.05) between different treatments compared to the respective control group.

In the present research, we measured the 11-KT concentration to partially assess the effect of methimazole-induced hypothyroidism on steroidogenesis, *in vivo* and *in vitro* (Figure 4A). Four treatment groups were studied in this experiment, including control, control + T4, methimazole and co-treatment (methimazole + T4) groups (Figure 4A). Animal exposure with T4 (control + T4) significantly reduced the plasma 11-KT level as compared to control levels (Figure 4B). Also, treatment with methimazole significantly reduced the plasma 11-KT concentration to almost undetectable level compared to the control level of over 40 ng/ml (Figure 4B). However, co-treatment with T4 significantly increased and nullified the methimazole-induced response to a level not significantly different from the control (Figure 4B).

We also measured the androgen (11-KT) release capacity of zebrafish testicular tissue using the *ex vivo* culture system (Figure 4A). In this experiment, we compared testis taken from control and those exposed to T4 (100 μg/L), methimazole (1 mM) and methimazole co-treated with T4. We tested the 11-KT release response into culture media following *in vitro* treatment for 18 h to recombinant zebrafish Fsh (100 ng/ml) (Figure 4A). In the basal medium condition, the treatments (T4, methimazole and methimazole + T4) did not change the basal androgen (11-KT) release capacity of zebrafish testicular tissue (Figure 4C). However, as expected, treatment with Fsh significantly increased the 11-KT level in the control group (Figure 4C). Also, treatment with T4 was responsive to Fsh but to a level not significantly different from the control (Figure 4C). In comparison, the isolated testis from methimazole-treated zebrafish was completely unresponsive to Fsh (Figure 4C). However, co-treatment with T4 restored the Fsh-induced response by increasing the 11-KT concentration to the level observed following treatment of the control group with Fsh alone (Figure 4C).

### 3.6 Transcript Levels of Selected Genes in the Brain and Pituitary

**FIGURE 5:**
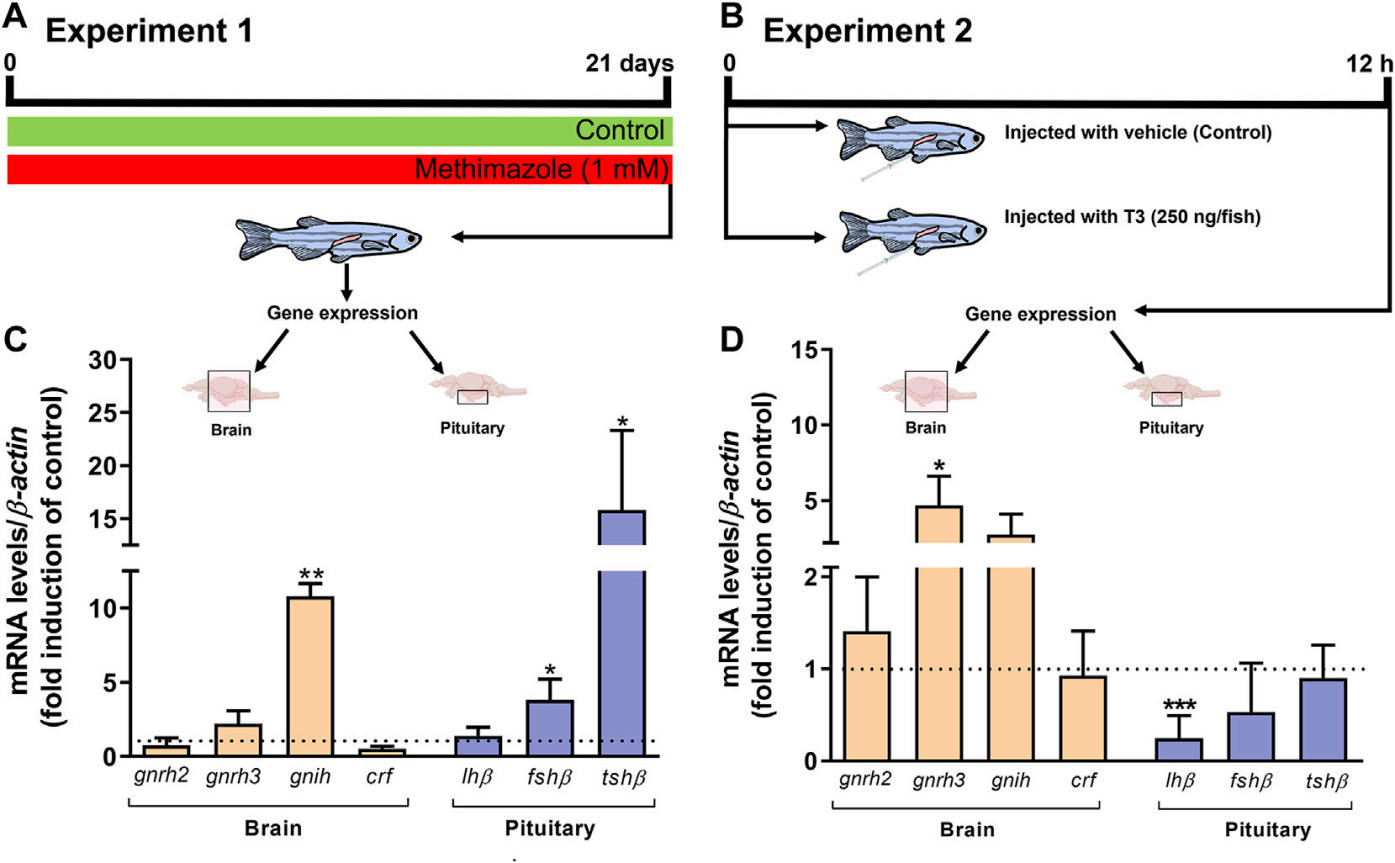
**(A)** Experiment 1: zebrafish adult males were exposed to methimazole-induced hypothyroidism for 3 weeks. **(B)** Experiment 2: zebrafish adult males were injected with 0 or 250 ng of T3/fish and tissue were collected 12 h post-injection. Relative mRNA levels of selected genes, including *gnrh2* and *gnrh3* (gonadotropin-releasing hormones), *gnih* (gonadotropin-inhibitory hormone), and *crf* (corticotropin-releasing hormone), expressed in the brain (*n* = 8), and *lhβ* (luteinizing hormone), *fshβ* (follicle-stimulating hormone), and *tshβ* (thyroid-stimulating hormone) expressed in the pituitary (*n* = 4 pools of 4 pituitaries for each pool) from the methimazole group **(C)** and 12 h post-injection with 0 and 250 ng of T3/fish **(D)**. Ct values were normalized with *β-actin* and expressed as relative values of control (no-treated fish) levels of expression. Bars represent mean ± SEM fold-change relative to the control, which is set at 1. Student unpaired *t*-test, *p < 0.05, **p < 0.01 and ***p < 0.001.

In this experiment, we measured brain transcript levels of a number of neurohypothalamic peptides, including *gnrh2* and *gnrh3*, *gnih*, and *crf*, as well as pituitary gonadotropin hormone subunits, *fshb*, *lhb*, and *tshb* in the methimazole-induced hypothyroidism in fish (Figure 5A,C). As shown in Figure 5C, we observed a significant increase in the *gnih* transcript level compared to the control shown as the dotted line. Transcript levels of the other neuropeptides measured including *gnrh2*, *gnrh3* and *crf* remained unchanged. In the same group of methimazole-treated fish, the results the pituitary glycoprotein hormone subunits, demonstrate significant increase in *fshb* mRNA and a massive increase in *tshb* transcript levels in the methimazole-induced hypothyroid fish (Figure 5C). No change was observed in the *lhb* transcript level (Figure 5C).

We also measured the same transcript levels following 12 h acute treatment with 250 ng of T3/fish *in vivo* (Figures 5B,D). In this experiment T3 injection did not significantly alter *gnih, gnrh2* and *crf,* but significantly increased the *gnrh3* transcript level (Figure 5D). In the same group of fish, acute treatment with 250 ng of T3/fish significantly reduced the pituitary *lhb* but was without effect on the *fshb* and *tshb* transcript levels (Figure 5D).

### 3.7 *In Vitro*: Effects of T3 on Zebrafish Spermatogenesis

**FIGURE 6:**
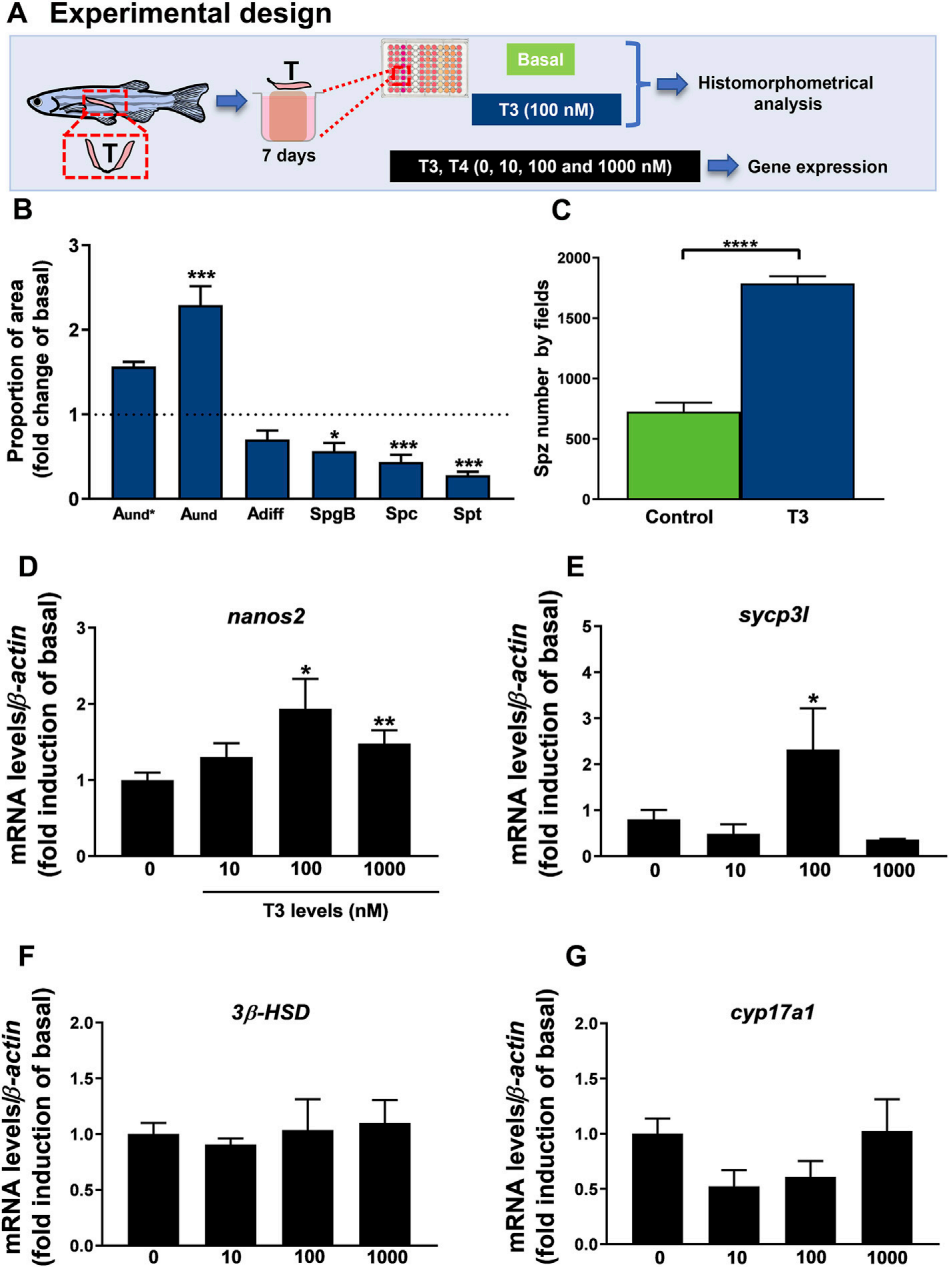
**(A)** Experimental design of histomorphometrical analysis of zebrafish testicular explants incubated for 7 days (long-term exposure) with T3 (100 nM) compared to the control (basal) and gene expression of relative mRNA levels of several selected genes in zebrafish testis incubated to different concentrations of T3 (0, 10, 100 e 1,000 nM) for 7 days. **(B)** Histomorphometrical analysis of testicular explants containing types A undifferentiated spermatogonia (A_und*,_ A_und_), type A differentiated spermatogonia (A_diff_), type B spermatogonia (SpgB), spermatocytes (Spc), and spermatids (Spt). Bars (mean ± SEM; *n* = 8) are expressed as fold-change relative to the untreated group (control) (dotted black line set at 1). **(C)** Spermatozoa number per field generated by using IMAGEJ Software from zebrafish explants incubated for 7 days with basal (L-15) and T3 (100 nM). Bars (mean ± SEM; *n* = 8). Student paired *t*-test, *p < 0.05 and ***p < 0.001 denote significant differences between control and treated fish. The selected genes *nanos2* **(D)**, *sycp3l* (synaptonemal complex protein 3) **(E)**, *3β-HSD* (3-beta (β)-hydroxysteroid dehydrogenase) **(F)**, and *cyp17a1* (17α-hydroxylase/17,20 lyase/17,20 desmolase) **(G)** were evaluated. Ct values were normalized with *β-actin* and expressed as relative values of basal levels of expression. Bars represent the mean ± SEM fold change (*n* = 8), relative to the control (basal), which is set at 1. Paired *t -test*, *p < 0.05, **p < 0.01.

In this research, we also examined the direct action of T3 at 100 nM on *ex vivo* culture of zebrafish testis for 7 days (Figure 6A) and results provide information on the proportion of germ cells in the cultured testis. Histomorphometrical evaluation of zebrafish testis revealed that treatment with T3 significantly stimulated the type A undifferentiated spermatogonia (A_und_) abundance with no effect on type A differentiated spermatogonia cells as compared to basal medium incubation (Figure 6B). In the same tissue, we observed a reduction in type B spermatogonia (SpgB) abundance (Figure 6B). As for meiotic and post-meiotic cells, treatment with T3 reduced the proportion of area occupied by Spc and Spt when compared to basal condition (Figure 6B). Interestingly, the number of spermatozoa was stimulated in zebrafish testis treated with T3 as clearly shown by the analysis of spermatozoa number by field (Figure 6C). In addition to these results, the effect of thyroid hormones on testicular gene expression was analyzed (Figures 6D–G) (Supplementary Figure S1). Several concentrations of T3 and T4 (10, 100 and 1,000 nM) were tested in zebrafish testis. T4 did not change the expression of any transcript (*nanos2*, *sycp3l*, *3β-HSD* and *cyp17a1*) (Supplementary Figure S1). On the other hand, expression analysis revealed that T3 (100 and 1,000 nM) increased *nanos2* (Figure 6D), and up-regulated *sycp3l* transcript levels at 100 nM (Figure 6E). Levels of *3β-HSD* and *cyp17a1* were also quantified and did not change within any levels of T3 (Figures 6F,G).

## 4 Discussion

This study demonstrated the importance of thyroid hormones as a factor controlling zebrafish spermatogenesis, using methimazole-induced hypothyroidism in fish as a model organism. Methimazole is a thionamide antithyroid drug, and it is known to block thyroid hormone synthesis by affecting the iodination process of tyrosine residues in the thyroglobulin (Abraham and Acharya, 2010; Carvalho and Dupuy, 2017). Methimazole acts through suppressing the thyroid peroxidase (TPO) activity which is responsible to catalyze the conversion of iodide to iodine for the production of T4 and T3. In addition to this effect, methimazole is rapidly metabolized by the liver and may cause hepatotoxicity and other potent side effects (Heidari et al., 2015). In this study, we could not rule out or monitor the side effects caused by methimazole treatment. However, the effects observed in zebrafish spermatogenesis are caused by thyroid hormone deficiency, and not due to methimazole side effect. We demonstrated that the defects observed in zebrafish spermatogenesis after methimazole treatment were completely recovered by adding T4 in the last week of methimazole treatment (see below). In addition, we confirmed that plasma T3 levels were significantly decreased following methimazole treatment when compared to control, and restored in the methimazole + T4 co-treatment. This data was also supported by histological analysis of thyroid follicles which displayed disturbed structure and less colloid production following methimazole exposure, while normal thyroid follicle histology and colloid production were found after co-treatment with T4. Another evidence is the testicular connexin (*cx43*), as it is the potential target for thyroid hormones (Poguet et al., 2003; Gilleron et al., 2006). Gilleron and collaborators (2006) demonstrated that propylthiouracil (PTU; another thyroid disruptor) decreases *cx43* mRNA levels in rat testes. In our study, the lower expression of *cx43* in the methimazole group indicates that methimazole treatment led to hypothyroidism in the male zebrafish. Altogether, this evidence confirmed that zebrafish testicular function and spermatogenesis are affected when lowering thyroid hormone levels, and the observed defects were nullified when plasma thyroid hormone levels were recovered to their basal levels.

Histomorphometric evaluation of zebrafish spermatogenesis demonstrated that methimazole-induced hypothyroidism remarkably increased the proportion area occupied by type A_und*_ (the most undifferentiated spermatogonia), type A_diff_ and type B spermatogonia, while the number of spermatozoa was decreased. Furthermore, our results demonstrate that the proportion of Spc and Spt were not affected in hypothyroid testis compared with the control animals. It has been well-established that spermatogenesis development is supported and regulated by gonadotropic hormones, FSH and LH which mainly target actions on somatic testicular cells such as Sertoli and Leydig cells (Planas et al., 1993; Campbell et al., 2003; Huhtaniemi and Themmen, 2005; García-López et al., 2010; Schulz et al., 2010; Flood et al., 2013). Likewise, thyroid hormones have also been shown to play an essential role in testicular physiology (Chiao et al., 2002; Cooke et al., 2004; Holsberger and Cooke, 2005; Wagner et al., 2008, 2009; Morais et al., 2013; Tovo-Neto et al., 2018). In rats, previous studies have reported that hypothyroidism decreases plasma levels of gonadotropins and reduces the number and size of gonadotrophs (Bruni et al., 1975; Ruiz et al., 1988), suggesting that the absence of thyroid hormones is associated with gonadal dysfunctions (Amin and El-Sheikh, 1977; Hernandez, 2018). Further, the level of relevant androgen (11-KT) that stimulates spermatogenesis decreased significantly in the plasma of zebrafish following treatment with methimazole. These results, in part, could explain the observed increase in different populations of spermatogonia (A_und*_, A_diff_ and B) and decrease in spermatozoa number, demonstrating the impact of thyroid hormone depletion on germ cell development and testicular function. Similar findings have been reported for male Japanese quail, wherein treatment with methimazole decreased body and testes weight as well as plasma levels of LH and testosterone (Weng et al., 2007). In this same study, the data showed decreased spermatogenesis in seminiferous tubules of the treatment group (Weng et al., 2007). Altogether, these results demonstrate that gonadal function is associated with normal thyroid action. Fluctuations in thyroid hormone levels can directly modulate gonadotropin actions and affect Sertoli and Leydig cell proliferation (Castañeda-Cortés et al., 2014), resulting in the impairment of spermatogenesis, which may be caused by low FSH level and delay of Sertoli cell maturation (Hernandez, 2018).

We also examined the effects of methimazole co-treated with T4. Our results demonstrate that co-treatment with T4 partially restored zebrafish spermatogenesis and spermatozoa production in the methimazole-induced hypothyroid fish. Interestingly, the administration of T4 to the hypothyroid group increased the proportion of meiotic (Spc) and post-meiotic (Spt) cells compared with the control and methimazole groups. These results support the hypothesis that thyroid hormones are involved, directly or indirectly, with meiosis entry in the zebrafish testis.

The observed transcript analysis in zebrafish testis is to some extent in agreement with histomorphometric results. With respect to the germ cell markers genes of zebrafish spermatogenesis, the results indicated that mRNA levels of *nanos2* (marker of type A undifferentiated spermatogonia) (Aoki et al., 2009) and *dazl* (marker of differentiation) (Chen et al., 2013) were greater in the methimazole-treated group, in agreement with the histomorphometry showing an accumulation of pre-meiotic cells (A_und*_, A_diff_ and B). The observed upregulation of these transcripts in the methimazole treated group suggest that euthyroid condition may be important for normal spermatogonial cell development in zebrafish. However, the level of *odf3a* (marker of spermatids) (Yano et al., 2008) was upregulated in the methimazole group, although the proportion of spermatids was not affected in the present study. The affected testicular functions did not always recover following co-treatment with T4 to counter hypothyroidism.

Other essential components of the thyroid hormones axis are the thyroid receptors (TRs), which are crucial for testis development and function (Valadares et al., 2008; Lema et al., 2009; Wajner et al., 2009; Dittrich et al., 2011). According to Habibi and collaborators (2012), TRs are expressed by some different cell types, and thyroid hormones have pleiotropic effects, including effects on the gonads. In the latter study in goldfish, *in vivo* and *in vitro*, thyroid hormones were shown to exert both direct and indirect actions on gonadal steroid synthesis, and steroid receptor expression in a seasonally dependent manner (Allan and Habibi, 2012; Habibi et al., 2012). In zebrafish testis, *thrα* was shown to be expressed in the Sertoli and Leydig cells, whereas *thrβ* expression was only observed in the Leydig cell (Morais et al., 2013). In the present study, methimazole-induced hypothyroidism stimulated *thrβ* transcript level, but increase in *thrα* was only significant in presence of T4 compared to control. Other investigators also reported that T3 treatment only elevated *thrβ* in the ovary and testis of *Pimephales promelas* (Lema et al., 2009). Similarly, *fshr* mRNA levels were also upregulated in hypothyroid co-treated with T4. These results suggest that chronic hyperthyroidism may alter thyroid hormone sensitivity and possibly alter other parameters not clear at the present time. Similarly, in rats, higher FSH-R mRNA levels were detected in hypothyroidism. It was suggested that the upregulated FSH-R mRNA level may have resulted from the elevated proportion of Sertoli cells in rats (Rao et al., 2003). Likewise, in zebrafish, the increase of *fshr* transcripts following co-treatment with T4 could be due to Sertoli cell proliferation. Morais and collaborators (2013) revealed that T3 stimulates *in vitro* Sertoli cell proliferation in zebrafish testes. Moreover, the same study showed that Sertoli cells express both *thrα* and *fshr*, and treatment with T3 potentiates Fsh *in vitro* actions in the zebrafish testis (Morais et al., 2013). This is also consistent with the observation that *fshr* transcript levels was stimulated by methimazole-induced hypothyroid fish co-treated with T4. These results support the view that thyroid hormones and gonadotropins stimulate spermatogenesis by stimulating gonadal androgen production, which in turn mediate the start of spermatogenesis.

Interestingly, mRNA expression of other key gonadal growth factors as *igf3* (Wang et al., 2008) and *amh* (Miura et al., 2002) did not change in the methimazole or methimazole co-treated with T4 groups. However, *gsdf* (Gautier et al., 2011) was upregulated in both groups. *gsdf* is expressed by Sertoli cells and exerts an essential role in the control of spermatogenesis, including germ cell proliferation and differentiation (Gautier et al., 2011; Yan et al., 2017).

Decreased thyroid hormones levels by methimazole in male zebrafish are associated with decreased 11-KT plasma levels. Previous reports have demonstrated that PTU treatment also decreases serum testosterone concentrations in other vertebrate species, such as rats (Chiao et al., 2000). In another study, exposure of *Clarias gariepinus* to thiourea, reduced androgen levels leading to testicular regression (Swapna et al., 2006; Swapna and Senthikumaran, 2007). This is consistent with the present study demonstrating lower 11-KT plasma levels in the methimazole group, in addition to lower levels of steroidogenic enzyme (*cyp17a1*) and androgen-sensitive gene (*insl3*) mRNA levels. These transcript levels remained the same even after co-treatment with T4. However, treatment of methimazole-induced hypothyroid fish effectively rescued 11-KT levels in the plasma which was correlated with the revival of spermatozoa number in zebrafish testis. Thus, impairing thyroid action within the physiological limit of restoration is a valid approach to explain the role of thyroid hormones in male zebrafish reproduction. Interestingly, Houbrechts et al. (2019) demonstrated the Dio2-knockout (KO) zebrafish (*dio2* ^*−/−*^) significantly reduced androgen (11-KT and testosterone) levels in the testis, and steroidogenesis and steroid signaling were similarly disturbed when compared with control group. This indicates that absence of thyroid hormones by goitrogen treatment or DIO2 KO, which suppresses thyroid hormone production, may act directly on the testis to repress steroidogenesis. These data also reveal that normal thyroid hormone levels are fundamental to normal reproduction in zebrafish. On the other hand, Song et al. (2021) have recently reported that knockout of *tshba* in zebrafish resulted in defective development of secondary sex characteristics, but did not affect the spermatogenesis, indicating that thyroid hormones may be not necessary to male germ cell development. According to our knowledge, we cannot compare the methimazole exposure with the knockout of *tshba* in zebrafish. These two models represent thyroid loss of function in different stages of life of zebrafish. Our model is a chronic exposure (21 days) in sexually mature males (3 months of age), while *tshb* knockout took place from fertilization until sexual maturation, i.e., along the entire development of zebrafish. In addition to that, we believe that our results are similar to the ones obtained in the knockout of *tshb* in zebrafish. Song and collaborators (2021) reported reproductive performance defects, but normal gonadal histology. However, no histomorphometry data (e.g., germ cell cyst composition) was performed in their study, and detailed analysis of the gonadal histology (Song et al., 2021) showed a reduced testicular lumen and apparently less spermatozoa in the *tshba−/−* as compared to wild type (*tshba+/+*)*.* Histomorphometric analysis of zebrafish *tshba−/−* testis should be investigated to address whether thyroid hormones are necessary for zebrafish spermatogenesis.

To address whether thyroid hormones can affect the expression of regulatory players of HPG axis in long-term or acute treatments, we performed two experiments: experiment 1 (methimazole exposure for 21 days—lowering of thyroid hormones) and experiment 2 (intracoelomic injection of T3 for 12 h) (Figure 5). In experiment 1, while transcript levels of gonadotropin-releasing hormones, *gnrh2* and *gnrh3* did not change, *gnih* mRNA levels were significantly elevated in the brain of animals after methimazole exposure. GnIH is a gonadotropin-inhibitory hormone and it has been shown to block gonadotropin secretion and reduce its plasma levels, therefore affecting reproduction (Tsutsui et al., 2017). In fish, GnIH orthologs have been described to exert either stimulatory or inhibitory actions on gonadotropin secretion depending on the species studied, season, and mode of treatment (Amano et al., 2006; Zhang et al., 2010; Moussavi et al., 2012; 2013; 2014; Branco et al., 2018; Fallah et al., 2019; Ma et al., 2020a, Ma et al., 2020b). In cyprinids, Zhang and collaborators (2010) have shown that zebrafish GnIH decreases plasma gonadotropin levels in goldfish. In mammals, Tsutsui et al. (2017) remarkably showed that hypothyroidism induced by PTU increases GnIH expression and reduces gonadotropins and plasma steroid levels in female mice. Altogether, this evidence can support that the decrease in plasma androgen level observed after methimazole treatment might be mediated by GnIH. As shown previously in goldfish, GnIH mediated inhibition of reproduction does not always correlate closely by inhibition of gonadotropin transcript levels due to uncoupling of release and synthesis. In this context, it was demonstrated that GnIH can increase gonadotropin subunit mRNA levels while reducing secretion of the hormones (Moussavi et al., 2012; Moussavi et al., 2013; Moussavi et al., 2014; Ma et al., 2020a, Ma et al., 2020b). Thus, the inhibition may be at the protein level, since *fshb* transcript level was increased in the pituitary of the methimazole-treated group as a compensatory/feedback mechanism. Similarly, Yoshiura et al. (1999) showed in goldfish that thiourea exposure for 2 weeks increased Fsh mRNA levels. A contributing factor could be due to feedback exerted by lower plasma 11-KT levels seen in these animals (Rojdmark et al., 1988; Trudeau et al., 1993; Habibi and Huggard-Nelson, 1998; Huggard-Nelson et al., 2002). There is increasing evidence that the effects of thyroid hormones as well as brain-pituitary-gonadal hormones are diverse, and change with season, mode of action, time-course, and concentration (Nelson and Habibi 2006, Nelson and Habibi 2008, Nelson and Habibi 2009; Nelson et al., 2011; Habibi et al., 2012; Moussavi et al., 2012, Moussavi et al., 2013, Moussavi et al., 2014; Nelson and Habibi 2016; Ma et al., 2020a, Ma et al., 2020b). This is reflected in the experiment 2 in which the acute treatment for 12 h with T3 injection resulted in a different effect than the ones observed in the chronic treatment with methimazole. In Experiment 2, injection of T3 increases *gnrh3* mRNA levels, which indicates that the HPG axis is stimulated/activated. As a consequence of the BPG stimulation, a negative feedback on gonadotropin mRNA levels is expected. This explains the reduced *lh* levels following T3 injection in Experiment 2. This is coherent with earlier study in goldfish showing reduction in gonadotropins and synthesis of gonadal steroids following 12 h injection with T3 (Nelson et al., 2010). Altogether these results demonstrate that the HPG axis is affected when altering thyroid hormone levels in long or acute treatments. However, we were not able to explain the differences of *lh* and *fsh* expression between the two Experiments/conditions. We believe that this difference might be due to distinct regulatory mechanisms between the two gonadotropins.

In addition to these data, we examined the actions of thyroid hormones on zebrafish spermatogenesis. Histomorphometrical analysis revealed an increase of type A undifferentiated spermatogonia (A_und_) proportion in T3 *in vitro* exposure (100 nM), which may reflect the up-regulation of *nanos2* (marker of type A undifferentiated spermatogonia) (100 and 1,000 nM of T3). The number of spermatozoa also increased. This data corroborates the histomorphometry of the zebrafish testes from *in vivo* exposures using methimazole and methimazole co-treated with T4, indicating, therefore, a direct action of thyroid hormones in the zebrafish spermatogenesis. Previously, Morais et al. (2013) reported that the mitotic indices of A_und_ and Sertoli cells were stimulated after T3 treatment. Likewise, Safian and collaborators (2016) reported that T3 improved the formation of new cysts of A_und_, accumulation of differentiated spermatogonia (A_diff_) and reduction of spermatogonia Morais et al. (2013) using lower dose of T3 (50 ng/ml) also demonstrated an increase of type A undifferentiated spermatogonia, which was corroborated by higher BrdU mitotic index of this cell type in T3-treated tissue. In this study, we also investigated the effects of T3 and T4 on gene expression. Several concentrations of T3 and T4 (10, 100 and 1,000 nM) were tested in zebrafish testis. Interestingly, T3 also stimulated *sycp3l* (meiotic marker) mRNA levels at 100 nM. Levels of *3β-HSD* and *cyp17a1* were also quantified and did not change within any level of T3 exposure. Another interesting result is that T4 does not exert any change on testicular gene expression, indicating that thyroid hormones act via T3 in zebrafish testis. In sum, this data support that thyroid hormone T3 stimulates zebrafish spermatogenesis by proliferation, differentiation, and meiosis. These results provide important data about the roles of thyroid hormones on brain-pituitary-testis and reinforce the interaction between reproductive and thyroid axes.

## 5 Conclusion

The present study clearly demonstrated that hypothyroidism induced by methimazole significantly impair zebrafish germ cell development by delaying differentiation and meiosis, and decreasing the number of spermatozoa as well as impairing the hypothalamic–pituitary axis. We also provide evidence that testicular function is dependent on thyroid hormones. Taken together, these results provide support for the hypothesis that thyroid hormones are essential for spermatogenesis and maintaining normal function of the hypothalamic–pituitary-gonadal axis in adult zebrafish.

## Data Availability

The original contributions presented in the study are included in the article/Supplementary Material, further inquiries can be directed to the corresponding author.

## Supplementary Material

Supplementary Figure S1
**(A)** Relative mRNA levels of selected genes expressed in zebrafish testis after incubated for 7 days to different concentrations of T4 (0, 10, 100 e 1,000 nM). The following genes were evaluated: *nanos2* **(B)**, *sycp3l* (synaptonemal complex protein 3) **(C)**, *3β-HSD* (3-beta (β)-hydroxysteroid dehydrogenase) **(D)**, and *cyp17a1* (17α-hydroxylase/17,20 lyase/17,20 desmolase) **(E)**. Ct values were normalized with *β-actin* and expressed as relative values of basal levels of expression. Bars represent the mean ± SEM fold change (*n* = 8), relative to the control (basal), which is set at 1. Paired *t -test*,

Supplementary Table S1 Primers used for gene expression studies (qPCR) (FW = Forward; RV = Reverse).

## References

[B1] Abraham P. Acharya S. (2010). Current and Emerging Treatment Options for Graves&rsquo; Hyperthyroidism. Tcrm 6, 29–40. 10.2147/tcrm.s5229 PMC2817786 20169034

[B2] Allan E. R. O. Habibi H. R. (2012). Direct Effects of Triiodothyronine on Production of Anterior Pituitary Hormones and Gonadal Steroids in Goldfish. Mol. Reprod. Dev. 79, 592–602. 10.1002/mrd.22066 22752970

[B3] Amano M. Moriyama S. Iigo M. Kitamura S. Amiya N. Yamamori K. (2006). Novel Fish Hypothalamic Neuropeptides Stimulate the Release of Gonadotrophins and Growth Hormone from the Pituitary of Sockeye Salmon. J. Endocrinol. 188, 417–423. 10.1677/joe.1.06494 16522722

[B4] Amin S. O. El-Sheikh A. S. (1977). Pituitary-testicular Function Changes in Hypo-And Hyperthyroid Male Rats. Cells Tissues Organs 98, 121–129. 10.1159/000144788 860635

[B5] Aoki Y. Nakamura S. Ishikawa Y. Tanaka M. (2009). Expression and Syntenic Analyses of FournanosGenes in Medaka. Zoological Sci. 26, 112–118. 10.2108/zsj.26.112 19341327

[B6] Blanton M. L. Specker J. L. (2007). The Hypothalamic-Pituitary-Thyroid (HPT) axis in Fish and its Role in Fish Development and Reproduction. Crit. Rev. Toxicol. 37, 97–115. 10.1080/10408440601123529 17364706

[B7] Branco G. S. Melo A. G. Ricci J. M. B. Digmayer M. De Jesus L. W. O. Habibi H. R. (2019). Effects of GnRH and the Dual Regulatory Actions of GnIH in the Pituitary Explants and Brain Slices of *Astyanax Altiparanae* Males. General Comp. Endocrinol. 273, 209–217. 10.1016/j.ygcen.2018.08.006 30098316

[B8] Bruni J. F. Dibbet J. A. Meites J. Meites J. (1975). Effects of Hyper- and Hypothyroidism on Serum LH and FSH Levels in Intact and Gonadectomized Male and Female Rats. Endocrinology 97, 558–563. 10.1210/endo-97-3-558 1175507

[B9] Campbell B. Dickey J. T. Swanson P. (2003). Endocrine Changes during Onset of Puberty in Male Spring Chinook Salmon, *Oncorhynchus tshawytscha*1. Biol. Reprod. 69, 2109–2117. 10.1095/biolreprod.103.020560 12930714

[B10] Carr J. A. Patiño R. (2011). The Hypothalamus-Pituitary-Thyroid axis in Teleosts and Amphibians: Endocrine Disruption and its Consequences to Natural Populations. General Comp. Endocrinol. 170, 299–312. 10.1016/j.ygcen.2010.06.001 20566362

[B11] Carvalho D. P. Dupuy C. (2017). Thyroid Hormone Biosynthesis and Release. Mol. Cell. Endocrinol. 458, 6–15. 10.1016/j.mce.2017.01.038 28153798

[B12] CastaÃ±eda CortÃ©s D. C. Langlois V. S. Fernandino J. I. (2014). Crossover of the Hypothalamic Pituitaryâ€"Adrenal/Interrenal, â€"Thyroid, and â€"Gonadal Axes in Testicular Development. Front. Endocrinol. 5, 1–11. 10.3389/fendo.2014.00139 PMC4145579 25221542

[B13] Chen S. X. Bogerd J. Schoonen N. E. Martijn J. De Waal P. P. Schulz R. W. (2013). A Progestin (17α,20β-Dihydroxy-4-Pregnen-3-One) Stimulates Early Stages of Spermatogenesis in Zebrafish. General Comp. Endocrinol. 185, 1–9. 10.1016/j.ygcen.2013.01.005 23360837

[B14] Chiao Y.-C. Cho W.-L. Wang P. S. (2002). Inhibition of Testosterone Production by Propylthiouracil in Rat Leydig Cells1. Biol. Reprod. 67, 416–422. 10.1095/biolreprod67.2.416 12135875

[B15] Chiao Y.-C. Lin H. Wang S.-W. Wang P. S. (2000). Direct Effects of Propylthiouracil on Testosterone Secretion in Rat Testicular Interstitial Cells. Br. J. Pharmacol. 130, 1477–1482. 10.1038/sj.bjp.0703444 10928947 PMC1572209

[B16] Chiba H. Amano M. Yamada H. Fujimoto Y. Ojima D. Okuzawa K. (2004). Involvement of Gonadotropin-Releasing Hormone in Thyroxine Release in Three Different Forms of Teleost Fish: Barfin Founder, Masu Salmon and Goldfish. Fish. Physiol. Biochem. 30, 267–273. 10.1007/s10695-005-8676-y

[B17] Cooke P. S. Holsberger D. R. Witorsch R. J. Sylvester P. W. Meredith J. M. TreinenChapin K. A. R. E. (2004). Thyroid Hormone, Glucocorticoids, and Prolactin at the Nexus of Physiology, Reproduction, and Toxicology. Toxicol. Appl. Pharmacol. 194, 309–335. 10.1016/j.taap.2003.09.016 14761686

[B18] Cooke P. S. Meisami E. (1991). Early Hypothyroidism in Rats Causes Increased Adult Testis and Reproductive Organ Size but Does Not Change Testosterone Levels*. Endocrinology 129, 237–243. 10.1210/endo-129-1-237 2055186

[B19] Cooke P. S. Zhao Y.-D. Bunick D. (1994). Triiodothyronine Inhibits Proliferation and Stimulates Differentiation of Cultured Neonatal Sertoli Cells: Possible Mechanism for Increased Adult Testis Weight and Sperm Production Induced by Neonatal Goitrogen Treatment1. Biol. Reprod. 51, 1000–1005. 10.1095/biolreprod51.5.1000 7531505

[B20] Cyr D. Eales J. G. (1996). Interrelationships between Thyroidal and Reproductive Endocrine Systems in Fish. Rev. Fish. Biol. Fish. 6, 165–200. 10.1007/BF00182342

[B21] de Franca L. R. Hess R. A. Cooke P. S. Russell L. D. (1995). Neonatal Hypothyroidism Causes Delayed Sertoli Cell Maturation in Rats Treated with Propylthiouracil: Evidence that the Sertoli Cell Controls Testis Growth. Anat. Rec. 242, 57–69. 10.1002/ar.1092420108 7604982

[B22] De Oña C. R. Obregón M. J. Del Rey F. E. De Escobar G. M. (1988). Developmental Changes in Rat Brain 5′-Deiodinase and Thyroid Hormones during the Fetal Period: The Effects of Fetal Hypothyroidism and Maternal Thyroid Hormones. Pediatr. Res. 24, 588–594. 10.1203/00006450-198811000-00010 3205610

[B23] Dittrich R. Beckmann M. W. Oppelt P. G. Hoffmann I. Lotz L. Kuwert T. (2011). Thyroid Hormone Receptors and Reproduction. J. Reproductive Immunol. 90, 58–66. 10.1016/j.jri.2011.02.009 21641659

[B24] Duarte-Guterman P. Navarro-Martín L. Trudeau V. L. (2014). Mechanisms of Crosstalk between Endocrine Systems: Regulation of Sex Steroid Hormone Synthesis and Action by Thyroid Hormones. General Comp. Endocrinol. 203, 69–85. 10.1016/j.ygcen.2014.03.015 24685768

[B25] Fallah H. P. Rodrigues M. S. Corchuelo S. Nóbrega R. H. Habibi H. R. (2020). Role of GnRH Isoforms in Paracrine/autocrine Control of Zebrafish (*Danio rerio*) Spermatogenesis. Endocrinology 161, 1–16. 10.1210/endocr/bqaa004 31930304

[B26] Fallah H. P. Tovo-Neto A. Yeung E. C. Nóbrega R. H. Habibi H. R. (2019). Paracrine/autocrine Control of Spermatogenesis by Gonadotropin-Inhibitory Hormone. Mol. Cell. Endocrinol. 492, 110440. 10.1016/j.mce.2019.04.020 31048005

[B27] Figueiredo A. F. A. Wnuk N. T. Tavares A. O. Miranda J. R. Hess R. A. de França L. R. (2019). Prepubertal PTU Treatment in Rat Increases Sertoli Cell Number and Sperm Production. Reproduction 158, 201–211. 10.1530/REP-19-0127 31163400

[B28] Flood D. E. K. Fernandino J. I. Langlois V. S. (2013). Thyroid Hormones in Male Reproductive Development: Evidence for Direct Crosstalk between the Androgen and Thyroid Hormone Axes. General Comp. Endocrinol. 192, 2–14. 10.1016/j.ygcen.2013.02.038 23524004

[B29] García-López A. de Jonge H. Nóbrega R. H. de Waal P. P. Van Dijk W. Hemrika W. (2010). Studies in Zebrafish Reveal Unusual Cellular Expression Patterns of Gonadotropin Receptor Messenger Ribonucleic Acids in the Testis and Unexpected Functional Differentiation of the Gonadotropins. Endocrinology 151, 2349–236060. 10.1210/en.2009-1227 20308533 PMC2869266

[B30] Gautier A. Sohm F. Joly J.-S. Le Gac F. Lareyre J.-J. (2011). The Proximal Promoter Region of the Zebrafish Gsdf Gene Is Sufficient to Mimic the Spatio-Temporal Expression Pattern of the Endogenous Gene in Sertoli and Granulosa Cells1. Biol. Reprod. 85, 1240–1251. 10.1095/biolreprod.111.091892 21816849

[B31] Gilleron J. Nebout M. Scarabelli L. Senegas-Balas F. Palmero S. Segretain D. (2006). A Potential Novel Mechanism Involving Connexin 43 Gap Junction for Control of Sertoli Cell Proliferation by Thyroid Hormones. J. Cell. Physiol. 209, 153–161. 10.1002/jcp.20716 16823880

[B32] Habibi H. R. Huggard D. L. (1998). Testosterone Regulation of Gonadotropin Production in Goldfish. Comp. Biochem. Physiology Part C Pharmacol. Toxicol. Endocrinol. 119, 339–344. 10.1016/s0742-8413(98)00022-x 9827006

[B33] Habibi H. R. Nelson E. R. Allan E. R. O. (2012). New Insights into Thyroid Hormone Function and Modulation of Reproduction in Goldfish. General Comp. Endocrinol. 175, 19–26. 10.1016/j.ygcen.2011.11.003 22100124

[B34] Heidari R. Niknahad H. Jamshidzadeh A. Eghbal M. A. Abdoli N. (2015). An Overview on the Proposed Mechanisms of Antithyroid Drugs-Induced Liver Injury. Adv. Pharm. Bull. 10.5681/apb.2015.001 PMC4352210 25789213

[B35] Hernandez A. (2018). Thyroid Hormone Role and Economy in the Developing Testis. Vitam. Horm. 106, 473–500. 10.1016/bs.vh.2017.06.005 29407445

[B36] Hess R. A. Cooke P. S. Bunick D. Kirby J. D. (1993). Adult Testicular Enlargement Induced by Neonatal Hypothyroidism Is Accompanied by Increased Sertoli and Germ Cell Numbers. Endocrinology 132, 2607–2613. 10.1210/endo.132.6.8504761 8504761

[B37] Holsberger D. R. Cooke P. S. (2005). Understanding the Role of Thyroid Hormone in Sertoli Cell Development: a Mechanistic Hypothesis. Cell. Tissue Res. 322, 133–140. 10.1007/s00441-005-1082-z 15856309

[B38] Houbrechts A. M. Van houcke J. Darras V. M. (2019). Disruption of Deiodinase Type 2 in Zebrafish Disturbs Male and Female Reproduction. J. Endocrinol. 241, 111–123. 10.1530/JOE-18-0549 30817317

[B39] Huggard-Nelson D. L. Nathwani P. S. Kermouni A. Habibi H. R. (2002). Molecular Characterization of LH-β and FSH-β Subunits and Their Regulation by Estrogen in the Goldfish Pituitary. Mol. Cell. Endocrinol. 188, 171–193. 10.1016/s0303-7207(01)00716-x 11911956

[B40] Huhtaniemi I. T. Themmen A. P. N. (2005). Mutations in Human Gonadotropin and Gonadotropin-Receptor Genes. Endo 26, 207–218. 10.1385/ENDO:26:3:207 16034174

[B41] Jacobs G. F. M. Michielsen R. P. A. Kühn E. R. (1988). Thyroxine and Triiodothyronine in Plasma and Thyroids of the Neotenic and Metamorphosed Axolotl *Ambystoma mexicanum*: Influence of TRH Injections. General Comp. Endocrinol. 70, 145–151. 10.1016/0016-6480(88)90103-7 3131185

[B42] Jansen H. T. Kirby J. D. Cooke P. S. Arambepola N. Iwamoto G. A. (2007). Impact of Neonatal Hypothyroidism on Reproduction in the Male Hamster, *Mesocricetus auratus* . Physiology Behav. 90, 771–781. 10.1016/j.physbeh.2006.12.017 17291550

[B43] Kang H. Kenealy T. M. Cohen R. E. (2020). The Hypothalamic-Pituitary-Gonadal axis and Thyroid Hormone Regulation Interact to Influence Seasonal Breeding in Green Anole Lizards (*Anolis carolinensis*). General Comp. Endocrinol. 292, 113446. 10.1016/j.ygcen.2020.113446 32126224

[B44] Larsen D. A. Swanson P. Dickey J. T. Rivier J. Dickhoff W. W. (1998). In VitroThyrotropin-Releasing Activity of Corticotropin-Releasing Hormone-Family Peptides in Coho Salmon,*Oncorhynchus kisutch* . General Comp. Endocrinol. 109, 276–285. 10.1006/gcen.1997.7031 9473372

[B45] Leal M. C. De Waal P. P. García-López Á. Chen S. X. Bogerd J. Schulz R. W. (2009). Zebrafish Primary Testis Tissue Culture: An Approach to Study Testis Function *Ex Vivo* . General Comp. Endocrinol. 162, 134–138. 10.1016/j.ygcen.2009.03.003 19298819

[B46] Lema S. C. Dickey J. T. Schultz I. R. Swanson P. (2009). Thyroid Hormone Regulation of mRNAs Encoding Thyrotropin β-subunit, Glycoprotein α-subunit, and Thyroid Hormone Receptors α and β in Brain, Pituitary Gland, Liver, and Gonads of an Adult Teleost, *Pimephales promelas* . J. Endocrinol. 202, 43–54. 10.1677/JOE-08-0472 19380459

[B47] Ma Y. Ladisa C. Chang J. P. Habibi H. R. (2020a). Multifactorial Control of Reproductive and Growth axis in Male Goldfish: Influences of GnRH, GnIH and Thyroid Hormone. Mol. Cell. Endocrinol. 500, 110629. 10.1016/j.mce.2019.110629 31678419

[B48] Ma Y. Ladisa C. Chang J. P. Habibi H. R. (2020b). Seasonal Related Multifactorial Control of Pituitary Gonadotropin and Growth Hormone in Female Goldfish: Influences of Neuropeptides and Thyroid Hormone. Front. Endocrinol. 11, 175. 10.3389/fendo.2020.00175 PMC7154077 32318022

[B49] Mackenzie D. S. Sokolowska M. Peter R. E. Breton B. (1987). Increased Gonadotropin Levels in Goldfish Do Not Result in Alterations in Circulating Thyroid Hormone Levels. General Comp. Endocrinol. 67, 202–213. 10.1016/0016-6480(87)90149-3 3114043

[B50] Mackenzie D. S. (1982). Stimulation of the Thyroid Gland of a Teleost Fish, Gillichthys Mirabilis, by Tetrapod Pituitary Glycoprotein Hormones. Comp. Biochem. Physiology Part A Physiology 72, 477–482. 10.1016/0300-9629(82)90111-6 6126293

[B51] Matta S. L. P. Vilela D. A. R. Godinho H. P. França L. R. (2002). The Goitrogen 6-N-Propyl-2-Thiouracil (PTU) Given during Testis Development Increases Sertoli and Germ Cell Numbers Per Cyst in Fish: the tilapia (*Oreochromis niloticus*) Model. Endocrinology 143, 970–978. 10.1210/endo.143.3.8666 11861520

[B52] Mendis-Handagama S. M. Siril Ariyaratne H. B. (2005). Leydig Cells, Thyroid Hormones and Steroidogenesis. Indian J. Exp. Biol. 43, 939–962. 16313060

[B53] Miura T. Miura C. Konda Y. Yamauchi K. (2002). Spermatogenesis-preventing Substance in Japanese Eel. Development 129, 2689–2697. 10.1242/dev.129.11.2689 12015296

[B54] Morais R. D. V. S. Nóbrega R. H. Gómez-González N. E. Schmidt R. Bogerd J. França L. R. (2013). Thyroid Hormone Stimulates the Proliferation of Sertoli Cells and Single Type A Spermatogonia in Adult Zebrafish (*Danio rerio*) Testis. Endocrinology 154, 4365–4376. 10.1210/en.2013-1308 24002037

[B55] Moussavi M. Wlasichuk M. Chang J. P. Habibi H. R. (2012). Seasonal Effect of GnIH on Gonadotrope Functions in the Pituitary of Goldfish. Mol. Cell. Endocrinol. 350, 53–60. 10.1016/j.mce.2011.11.020 22155567

[B56] Moussavi M. Wlasichuk M. Chang J. P. Habibi H. R. (2013). Seasonal Effect of Gonadotrophin Inhibitory Hormone on Gonadotrophin-Releasing Hormone-Induced Gonadotroph Functions in the Goldfish Pituitary. J. Neuroendocrinol. 25, 506–516. 10.1111/jne.12024 23331955

[B57] Moussavi M. Wlasichuk M. Chang J. P. Habibi H. R. (2014). Seasonal Effects of GnIH on Basal and GnRH-Induced Goldfish Somatotrope Functions. J. Endocrinol. 223, 191–202. 10.1530/joe-14-0441 25319842

[B58] Mukhi S. Torres L. Patiño R. (2007). Effects of Larval-Juvenile Treatment with Perchlorate and Co-treatment with Thyroxine on Zebrafish Sex Ratios. General Comp. Endocrinol. 150, 486–494. 10.1016/j.ygcen.2006.11.013 17196199

[B59] Nelson E. R. Allan E. R. O. Pang F. Y. Habibi H. R. (2011). Auto-regulation of Thyroid Hormone Receptors in the Goldfish Ovary and Testis. General Comp. Endocrinol. 172, 50–55. 10.1016/j.ygcen.2010.12.017 21187097

[B60] Nelson E. R. Allan E. R. O. Pang F. Y. Habibi H. R. (2010). Thyroid Hormone and Reproduction: Regulation of Estrogen Receptors in Goldfish Gonads. Mol. Reprod. Dev. 77, 784–794. 10.1002/mrd.21219 20722048

[B61] Nelson E. R. Habibi H. R. (2006). Molecular Characterization and Sex-Related Seasonal Expression of Thyroid Receptor Subtypes in Goldfish. Mol. Cell. Endocrinol. 253, 83–95. 10.1016/j.mce.2006.05.003 16777315

[B62] Nelson E. R. Habibi H. R. (2008). Seasonal-related Homologous Regulation of Goldfish Liver Estrogen Receptor Subtypes. Cybium 32, 248–249. 10.26028/cybium/2008-322SP-124

[B63] Nelson E. R. Habibi H. R. (2016). Thyroid Hormone Regulates Vitellogenin by Inducing Estrogen Receptor Alpha in the Goldfish Liver. Mol. Cell. Endocrinol. 436, 259–267. 10.1016/j.mce.2016.08.045 27585488

[B64] Nelson E. R. Habibi H. R. (2009). Thyroid Receptor Subtypes: Structure and Function in Fish. General Comp. Endocrinol. 161, 90–96. 10.1016/j.ygcen.2008.09.006 18840444

[B65] Nóbrega R. H. Greebe C. D. Van De Kant H. Bogerd J. de França L. R. Schulz R. W. (2010). Spermatogonial Stem Cell Niche and Spermatogonial Stem Cell Transplantation in Zebrafish. PLoS One 5, e12808. 10.1371/journal.pone.0012808 20862221 PMC2942835

[B66] Orozco A. Valverde-R C. Olvera A. García-G C. (2012). Iodothyronine Deiodinases: a Functional and Evolutionary Perspective. J. Endocrinol. 215, 207–219. 10.1530/JOE-12-0258 22872760

[B67] Pankhurst N. W. (2016). “Reproduction and Development,” in Fish Physiology – Biology of Stress in Fish. Editors Schreck C. B. Tort L. Farrell A. Brauner C. (San Diego, CA: Academic Press), 295–331. 10.1016/b978-0-12-802728-8.00008-4

[B68] Patiño R. Wainscott M. R. Cruzli E. I. Balakrishnan S. Mcmurry C. Blazer V. S. (2003). Effects of Ammonium Perchlorate on the Reproductive Performance and Thyroid Follicle Histology of Zebrafish. Environ. Toxicol. Chem. 22, 1115–1121. 12729222

[B69] Planas J. V. Swanson P. Dickhoff W. W. (1993). Regulation of Testicular Steroid Production *In Vitro* by Gonadotropins (GTH I and GTH II) and Cyclic AMP in Coho Salmon (*Oncorhynchus kisutch*). General Comp. Endocrinol. 91, 8–24. 10.1006/gcen.1993.1099 8405894

[B70] Poguet A.-L. Legrand C. Feng X. u. Yen P. M. Meltzer P. Samarut J. (2003). Microarray Analysis of Knockout Mice Identifies Cyclin D2 as a Possible Mediator for the Action of Thyroid Hormone during the Postnatal Development of the Cerebellum. Dev. Biol. 254, 188–199. 10.1016/s0012-1606(02)00039-8 12591240

[B71] Rao J. N. Liang J. Y. Chakraborti P. Feng P. (2003). Effect of Thyroid Hormone on the Development and Gene Expression of Hormone Receptors in Rat Testes *In Vivo* . J. Endocrinol. Investig. 26, 435–443. 10.1007/BF03345199 12906371

[B72] Rodrigues M. S. Fallah H. P. Zanardini M. Malafaia G. Habibi H. R. Nóbrega R. H. (2021). Interaction between Thyroid Hormones and Gonadotropin Inhibitory Hormone in *Ex Vivo* Culture of Zebrafish Testis: An Approach to Study Multifactorial Control of Spermatogenesis. Mol. Cell. Endocrinol. 532, 111331. 10.1016/j.mce.2021.111331 34038752

[B73] Röjdmark S. Berg A. Kallner G. (1988). Hypothalamic-pituitary-testicular axis in Patients with Hyperthyroidism. Horm. Res. 29, 185–190. 10.1159/000181000 3146542

[B74] Roy P. Datta M. Dasgupta S. Bhattacharya S. (2000). Gonadotropin-Releasing Hormone Stimulates Thyroid Activity in a Freshwater Murrel, *Channa gachua* (Ham.), and Carps, Catla Catla (Ham.) and Cirrhinus Mrigala (Ham.). General Comp. Endocrinol. 117, 456–463. 10.1006/gcen.1999.7432 10764556

[B75] Safian D. Morais R. D. V. S. Bogerd J. Schulz R. W. (2016). Igf Binding Proteins Protect Undifferentiated Spermatogonia in the Zebrafish Testis against Excessive Differentiation. Endocrinology 157, 4423–4433. 10.1210/en.2016-1315 27689414

[B76] Schulz R. W. de França L. R. Lareyre J.-J. Legac F. Chiarini-Garcia H. Nobrega R. H. (2010). Spermatogenesis in Fish. General Comp. Endocrinol. 165, 390–411. 10.1016/j.ygcen.2009.02.013 19348807

[B77] Sharma P. Patiño R. (2013). Regulation of Gonadal Sex Ratios and Pubertal Development by the Thyroid Endocrine System in Zebrafish (*Danio rerio*). General Comp. Endocrinol. 184, 111–119. 10.1016/j.ygcen.2012.12.018 23337033

[B78] Sharma P. Tang S. Mayer G. D. Patiño R. (2016). Effects of Thyroid Endocrine Manipulation on Sex-Related Gene Expression and Population Sex Ratios in Zebrafish. General Comp. Endocrinol. 235, 38–47. 10.1016/j.ygcen.2016.05.028 27255368

[B79] Siril Ariyaratne H. B. Ian Mason J. Mendis-Handagama S. M. L. C. (2000). Effects of Thyroid and Luteinizing Hormones on the Onset of Precursor Cell Differentiation into Leydig Progenitor Cells in the Prepubertal Rat Testis1. Biol. Reprod. 63, 898–904. 10.1095/biolreprod63.3.898 10952937

[B80] Song J. Lu Y. Cheng X. Shi C. Lou Q. Jin X. (2021). Functions of the Thyroid-Stimulating Hormone on Key Developmental Features Revealed in a Series of Zebrafish Dyshormonogenesis Models. Cells. 10.3390/cells10081984 PMC8391828 34440752

[B81] Swapna I. Rajasekhar M. Supriya A. Raghuveer K. Sreenivasulu G. Rasheeda M. K. (2006). Thiourea-induced Thyroid Hormone Depletion Impairs Testicular Recrudescence in the Air-Breathing Catfish, *Clarias gariepinus* . Comp. Biochem. Physiology Part A Mol. Integr. Physiology 144, 1–10. 10.1016/j.cbpa.2006.01.017 16564715

[B82] Swapna I. Senthilkumaran B. (2007). Thyroid Hormones Modulate the Hypothalamo-Hypophyseal-Gonadal axis in Teleosts: Molecular Insights. Fish. Physiol. Biochem. 33, 335–345. 10.1007/s10695-007-9165-2

[B83] Teerds K. J. de Rooij D. G. De Jong F. H. Van Haaster L. H. (1998). Development of the Adult-type Leydig Cell Population in the Rat Is Affected by Neonatal Thyroid Hormone Levels1. Biol. Reprod. 59, 344–350. 10.1095/biolreprod59.2.344 9687306

[B84] Tousson E. Ali E. M. M. Ibrahim W. Mansour M. A. (2011). Proliferating Cell Nuclear Antigen as a Molecular Biomarker for Spermatogenesis in PTU-Induced Hypothyroidism of Rats. Reprod. Sci. 18, 679–686. 10.1177/1933719110395401 21273639

[B85] Tovo-Neto A. da Silva Rodrigues M. Habibi H. R. Nóbrega R. H. (2018). Thyroid Hormone Actions on Male Reproductive System of Teleost Fish. General Comp. Endocrinol. 265, 230–236. 10.1016/j.ygcen.2018.04.023 29678724

[B86] Tovo-Neto A. Martinez E. R. M. Melo A. G. Doretto L. B. Butzge A. J. Rodrigues M. S. (2020). Cortisol Directly Stimulates Spermatogonial Differentiation, Meiosis, and Spermiogenesis in Zebrafish (*Danio rerio*) Testicular Explants. Biomolecules 10, 429. 10.3390/biom10030429 PMC7175196 32164184

[B87] Trudeau V. L. Murthy C. K. Habibi H. R. Sloley B. D. Peter R. E. (1993). Effects of Sex Steroid Treatments on Gonadotropin-Releasing Hormone-Stimulated Gonadotropin Secretion from the Goldfish Pituitary1. Biol. Reprod. 48, 300–307. 10.1095/biolreprod48.2.300 8382536

[B88] Tsutsui K. Son Y. L. Kiyohara M. Miyata I. (2017). Discovery of GnIH and its Role in Hypothyroidism-Induced Delayed Puberty. Endocrinology 159, 62–68. 10.1210/en.2017-00300 28938445

[B89] ValadareS N. F. Polikarpov I. Garratt R. C. (2008). Ligand Induced Interaction of Thyroid Hormone Receptor Beta with its Coregulators. J. Steroid Biochem. Mol. Biol. 112, 205–212. 10.1016/j.jsbmb.2008.10.006 19000767

[B90] Van Der Ven L. T. M. Van Den Brandhof E.-J. Vos J. H. Power D. M. Wester P. W. (2006). Effects of the Antithyroid Agent Propylthiouracil in a Partial Life Cycle Assay with Zebrafish. Environ. Sci. Technol. 40, 74–81. 10.1021/es050972c 16433335

[B91] Wagner M. S. Wajner S. M. Maia A. L. (2009). Is There a Role for Thyroid Hormone on Spermatogenesis? Microsc. Res. Tech. 72, 796–808. 10.1002/jemt.20759 19637394

[B92] Wagner M. S. Wajner S. M. Maia A. L. (2008). The Role of Thyroid Hormone in Testicular Development and Function. J. Endocrinol. 199, 351–365. 10.1677/JOE-08-0218 18728126 PMC2799043

[B93] Wajner S. M. Wagner M. S. Maia A. L. (2009). Clinical Implications of Altered Thyroid Status in Male Testicular Function. Arq. Bras. Endocrinol. Metab. 53, 976–982. 10.1590/S0004-27302009000800011 20126850

[B94] Wang D.-S. Jiao B. Hu C. Huang X. Liu Z. Cheng C. H. K. (2008). Discovery of a Gonad-specific IGF Subtype in Teleost. Biochem. Biophysical Res. Commun. 367, 336–341. 10.1016/j.bbrc.2007.12.136 18166148

[B95] Weng Q. Saita E. Watanabe G. Takahashi S. Sedqyar M. Suzuki A. K. (2007). Effect of Methimazole-Induced Hypothyroidism on Adrenal and Gonadal Functions in Male Japanese Quail (*Coturnix japonica*). J. Reproduction Dev. 53, 1335–1341. 10.1262/jrd.19081 17965543

[B96] Xie X. Nóbrega R. Pšenička M. (2020). Spermatogonial Stem Cells in Fish: Characterization, Isolation, Enrichment, and Recent Advances of *In Vitro* Culture Systems. Biomolecules 10, 644. 10.3390/biom10040644 PMC7226347 32331205

[B97] Yan Y.-L. Desvignes T. Bremiller R. Wilson C. Dillon D. High S. (2017). Gonadal Soma Controls Ovarian Follicle Proliferation through Gsdf in Zebrafish. Dev. Dyn. 246, 925–945. 10.1002/dvdy.24579 28856758 PMC5761338

[B98] Yano A. Suzuki K. Yoshizaki G. (2008). Flow-cytometric Isolation of Testicular Germ Cells from Rainbow Trout (*Oncorhynchus mykiss*) Carrying the Green Fluorescent Protein Gene Driven by Trout Vasa Regulatory Regions. Biol. Reprod. 78, 151–158. 10.1095/biolreprod.107.064667 17901070

[B99] Yoshiura Y. Sohn Y. C. Munakata A. Kobayashi M. Aida K. (1999). Molecular Cloning of the cDNA Encoding the β Subunit of Thyrotropin and Regulation of its Gene Expression by Thyroid Hormones in the Goldfish*, Carassius auratus* . Fish. Physiol. Biochem. 21, 201–210. 10.1023/a:1007884527397

[B100] Zhang Y. Li S. Liu Y. Lu D. Chen H. Huang X. (2010). Structural Diversity of the Gnih/gnih Receptor System in Teleost: Its Involvement in Early Development and the Negative Control of LH Release. Peptides 31, 1034–1043. 10.1016/j.peptides.2010.03.003 20226824

