# Supplementary figures and images for: Thyroid Hormones Deficiency Impairs Male Germ Cell Development: A Cross Talk Between Hypothalamic-Pituitary-Thyroid, and—Gonadal Axes in Zebrafish
Source: Front Cell Dev Biol. 2022 May 12;10:865948. doi: 10.3389/fcell.2022.865948 (PMC9133415; doi:10.3389/fcell.2022.865948)

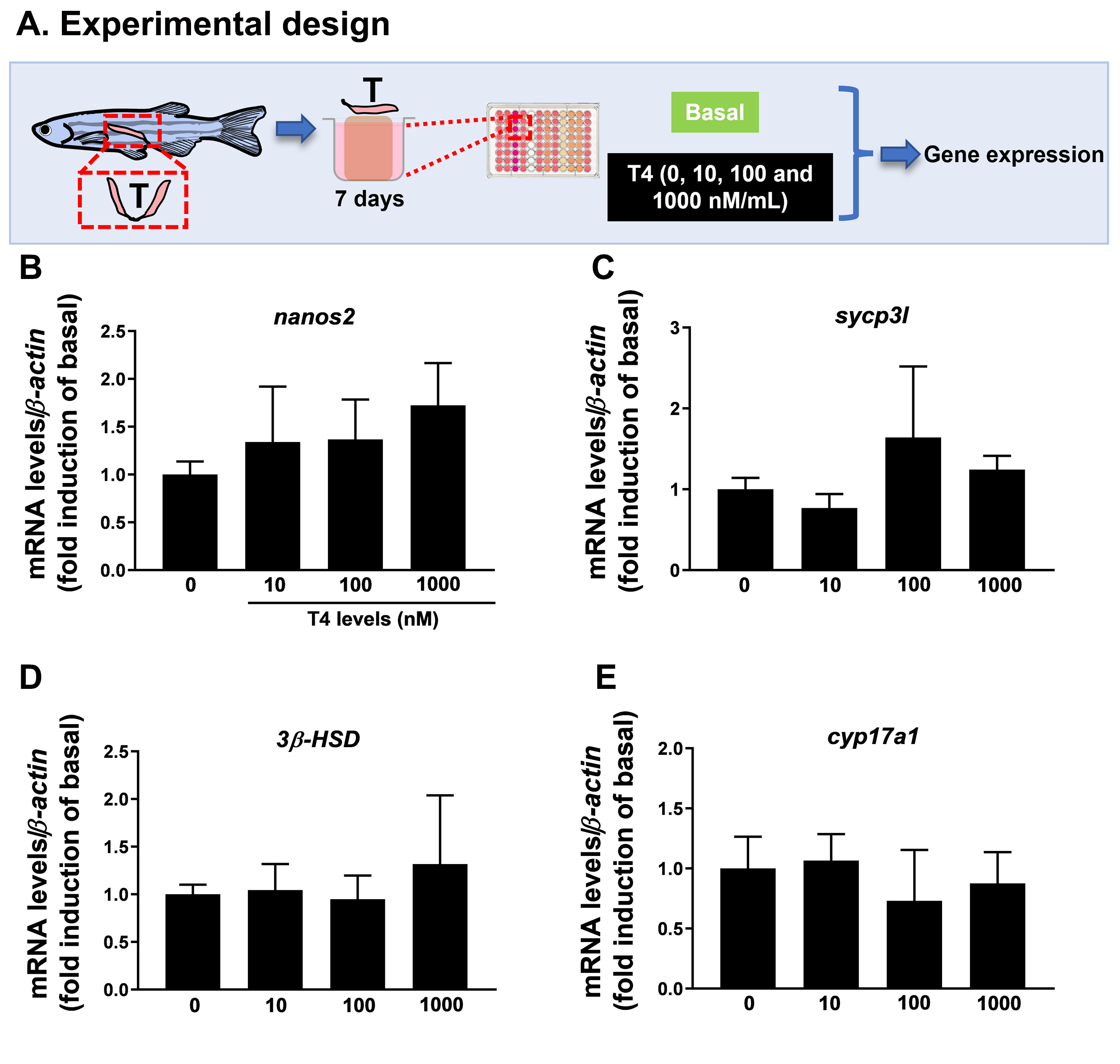

Supplement: Supplementary file 1 [file Image1.tif]
